# A novel classifier of radiographic knee osteoarthritis for use on knee DXA images is predictive of joint replacement in UK Biobank

**DOI:** 10.1093/rap/rkaf009

**Published:** 2025-01-20

**Authors:** Rhona A Beynon, Fiona R Saunders, Raja Ebsim, Benjamin G Faber, Mijin Jung, Jennifer S Gregory, Claudia Lindner, Richard M Aspden, Nicholas C Harvey, Timothy Cootes, Jonathan H Tobias

**Affiliations:** Musculoskeletal Research Unit, Bristol Medical School, University of Bristol, Bristol, UK; Centre for Arthritis and Musculoskeletal Health, University of Aberdeen, Aberdeen, UK; Division of Informatics, Imaging and Data Sciences, School of Health Sciences, University of Manchester, Manchester, UK; Musculoskeletal Research Unit, Bristol Medical School, University of Bristol, Bristol, UK; Medical Research Council Integrative Epidemiology Unit, University of Bristol, Bristol, UK; Musculoskeletal Research Unit, Bristol Medical School, University of Bristol, Bristol, UK; Centre for Arthritis and Musculoskeletal Health, University of Aberdeen, Aberdeen, UK; Division of Informatics, Imaging and Data Sciences, School of Health Sciences, University of Manchester, Manchester, UK; Centre for Arthritis and Musculoskeletal Health, University of Aberdeen, Aberdeen, UK; Medical Research Council Lifecourse Epidemiology Centre, University of Southampton, Southampton, UK; NIHR Southampton Biomedical Research Centre, University of Southampton and University Hospital Southampton NHS Foundation Trust, Southampton, UK; Division of Informatics, Imaging and Data Sciences, School of Health Sciences, University of Manchester, Manchester, UK; Musculoskeletal Research Unit, Bristol Medical School, University of Bristol, Bristol, UK; Medical Research Council Integrative Epidemiology Unit, University of Bristol, Bristol, UK

**Keywords:** knee osteoarthritis, radiographic osteoarthritis, DXA

## Abstract

**Objectives:**

DXA scans may offer a novel means of evaluating radiographic knee OA (rKOA) in large population studies and through opportunistic screening. We aimed to develop and apply a semi-automated method for assessing rKOA using ≈20 000 knee DXA images from UK Biobank (UKB) and assess its face validity by checking for expected relationships with clinical outcomes.

**Methods:**

Right knee DXA scans were manually annotated for osteophytes to derive corresponding grades. Joint space narrowing (JSN) grades in the medial joint compartment were determined from automatically measured minimum joint space width. Overall rKOA grade (0–4) was determined by combining osteophyte and JSN grades. Logistic regression was employed to investigate the associations of osteophyte, JSN and rKOA grades with knee pain and hospital-diagnosed KOA. Cox proportional hazards modelling was used to examine the associations of these variables with risk of subsequent total knee replacement (TKR).

**Results:**

Of the 19 595 participants included (mean age 63.7 years), 19.5% had rKOA grade ≥1 (26.1% female, 12.5% male). Grade ≥1 osteophytes and grade ≥1 JSN were associated with knee pain, hospital-diagnosed KOA and TKR. Higher rKOA grades were linked to stronger associations with these clinical outcomes, with the most pronounced effects observed for TKR. Hazard ratios for the association of rKOA grades with TKR were 3.28, 8.75 and 28.63 for grades 1, 2 and 3–4, respectively.

**Conclusions:**

Our DXA-derived measure of rKOA demonstrated a progressive relationship with clinical outcomes. These findings support the use of DXA for classifying rKOA in large epidemiological studies and in future population-based screening.

Key messages

- Radiographic knee osteoarthritis (rKOA) can be semi-automatically derived from DXA images.
- DXA-derived rKOA shows expected relationships with clinical outcomes of KOAs.
- DXA imaging presents a viable method for classifying rKOA in large-scale epidemiological research.

## Introduction

Knee osteoarthritis (KOA) is the most common form of osteoarthritis (OA), affecting 5.4 million people in the UK alone [1]. Annually, this results in ≈100 000 knee replacements being performed [2], with demand for these procedures expected to increase by nearly 40% by 2060 [3]. Diagnosis of KOA is primarily based on clinical symptoms, with persistent knee pain being the most common. Radiographically, KOA displays distinctive features such as osteophyte formation, joint space narrowing (JSN), subchondral sclerosis and cysts. These features have been integrated into grading systems for use in epidemiological studies, including the widely used Kellgren–Lawrence (KL) grading system [4], which classifies KOA severity into five grades ranging from 0 for ‘normal’ to 4 for ‘severe’. Typically, a KL grade ≥2, indicating the presence of a definite osteophyte and possible JSN, is used to define radiographic KOA (rKOA) in research studies [5]. However, applying this approach to large epidemiological studies is challenging due to its time-consuming and subjective nature [5–9]. As a result, there is growing interest in developing computer-aided techniques to enhance reliability and reduce the time required to derive these grades [10–15].

To date, large-scale epidemiological studies of OA have primarily relied on plain radiographs (X-rays), the modality for which the KL scoring system was developed. However, DXA imaging has recently emerged as a viable alternative [16]. Originally developed for measuring BMD at the hip and spine, DXA is now widely used in osteoporosis diagnosis. Advances in DXA technology have greatly enhanced its resolution, enabling the visualisation of joint features such as osteophytes and facilitating the measurement of JSN [16]. The very low radiation exposure associated with DXA devices [17] makes them well-suited for large-scale epidemiological studies, where repeated scans and longitudinal follow-up are valuable for examining rKOA progression. Moreover, the routine use of DXA in osteoporosis assessment provides a unique opportunity for complementary assessment of rKOA, particularly through opportunistic screening.

UK Biobank (UKB), a large prospective cohort study, is acquiring hip and knee DXA images from 100 000 participants [18]. A proof-of-concept study involving 40 000 of these hip DXA scans suggested that DXA images can be used to accurately classify hip OA; robust associations were observed between grades 2–4 of radiographic hip OA (rHOA) and various clinical outcomes, including a nearly 60-fold greater likelihood of requiring total hip replacement in individuals with rHOA grade 4 [19]. While similar studies for the knee are lacking, a previous study using knee DXA scans from UKB suggested that a DXA-derived imaging biomarker for knee shape, derived from a statistical shape model, could predict the need for total knee replacement (TKR) [20].

The primary objectives of this study were to develop a semi-automated method for classifying rKOA using DXA scans, to apply this method to a large dataset of images from UKB and to evaluate its face validity by examining its relationship with clinically important KOA outcomes.

## Methods

### Population

This study included participants from the UKB Extended Imaging Study, a subset of the larger UKB cohort. UKB enrolled ≈500 000 participants ages 40–69 years from across the UK between 2006 and 2010, collecting extensive health and lifestyle data. The Extended Imaging Study, initiated in 2014, aimed to collect medical imaging data, including DXA scans, from 100 000 participants [18]. UKB has full ethical approval from the National Information Governance Board for Health and Social Care and the North-West Multi-Centre Research Ethics Committee (11/NW/0382). All UKB participants provided consent, including permission for their health to be followed up through linkage to health-related records. This study was approved by the UKB under application number 17295.

### DXA-based measures of knee osteoarthritis

#### DXA-based scoring of osteophytes and JSN

High-resolution knee DXA scans were acquired using a Lunar iDXA scanner (GE Healthcare, Madison, WI, USA), with participants lying in a supine position. A machine-learning algorithm based on random forest regression voting (BoneFinder, University of Manchester [21]), initially trained on ≈7000 manually annotated left knee DXA images, placed 129 points along the bone contours of the distal femur, proximal tibia, proximal fibula and superior patella, excluding osteophytes. Details of this methodology have been published previously [20]. The present study is based on a selection of ≈20 000 randomly selected right knee DXA images with automated point placement checked by trained annotators (R.B. and F.S.).

**Figure 1.**
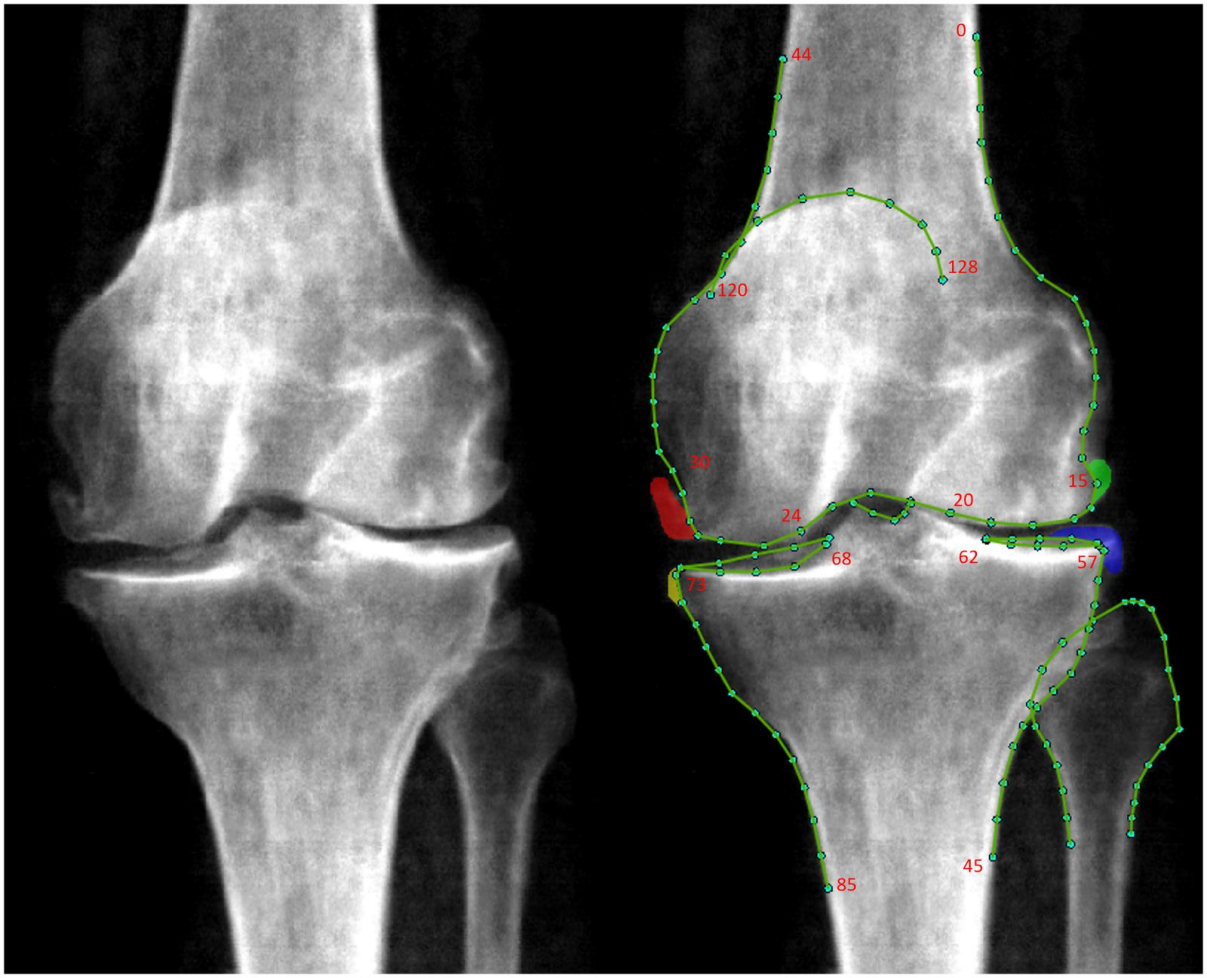
An example DXA scan with osteophytes marked up. The left panel displays a DXA image of the knee without annotations, while the right panel shows the same DXA image with osteophytes manually shaded. Osteophytes are indicated by colours corresponding to their locations: red for the medial femur, green for the lateral femur, yellow for the medial tibia and blue for the lateral tibia. The mJSW was measured at specific points in the medial and lateral compartments. For the distal femur, mJSW was measured between medial points 24–30 and lateral points 15–20. For the proximal tibia, mJSW was measured between medial points 68–73 and lateral points 57–62

At the time point placement was checked, each image was also evaluated for the presence of medial and lateral femoral and tibial osteophytes using a previously developed DXA-based atlas (created with input from D.W.) [20]. During training, interobserver repeatability between annotators (R.B. and F.S.) was assessed on a random sample of 200 images, demonstrating good agreement (κ = 0.68). If osteophytes were present, they were manually shaded (Fig. 1), and the osteophyte area (mm^2^) was calculated using a custom tool (University of Manchester). To ensure objectivity, the annotators were blinded to KOA outcomes during this process. In cases of uncertainty, images were jointly reviewed by the annotators and if consensus could not be reached, a final opinion was sought from J.T. (professor of rheumatology). Osteophytes were then automatically graded on a scale of 0–3 based on area thresholds derived from manual grading (Supplementary Data S1, available at *Rheumatology Advances in Practice* online).

The minimum joint space width (mJSW) of the medial joint compartment was automatically measured between predefined points (Fig. 1) using a custom Python 3.0 script (https://www.python.org/). Medial JSN grades were assigned based on the mJSW measurements: JSN grade 0 for mJSW >3 mm; grade 1 for mJSW >2.5–≤3 mm; grade 2 for mJSW >2–≤2.5 mm; and grade 3 for mJSW <2 mm. The medial joint compartment was selected due to its common involvement in primary KOA, with preliminary analyses indicating it as the most reliable predictor of clinical outcomes.

#### Generation of rKOA grades

**Figure 2.**
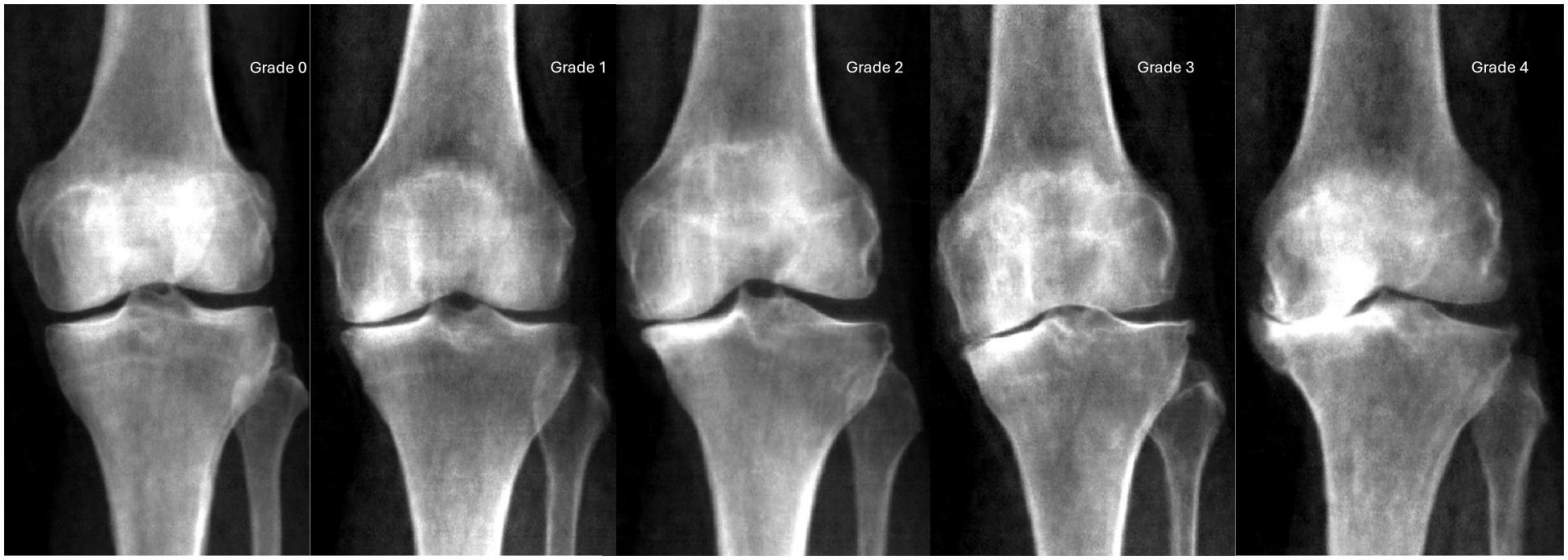
Example DXA scans representing each grade of rKOA. rKOA grades were generated by integrating data on osteophyte grades and JSN grades. The images show progression from grade 0 to grade 4, demonstrating increasing severity of OA changes. Grade 0 indicates no radiographic features of OA, while grades 1–4 show progressively more significant JSN and osteophyte formation

Overall rKOA grades were determined by integrating osteophyte and JSN grades. Subchondral sclerosis and cysts were not considered because they were rarely observed. Four osteophyte sites were assessed, each graded on a scale of 0–3, resulting in a total possible score of 12. To adjust for their relative contribution, each site’s grade was multiplied by 0.5, resulting in a maximum combined osteophyte score of 6. This score was then added to the JSN total, resulting in a maximum sum score of 9 (see Supplementary Table S1, available at *Rheumatology Advances in Practice* online). To provide a 5-point overall rKOA grade (similar to the KL radiograph grading system), we used the following cut-offs: rKOA grade 0, sum score = 0; grade 1, >0–≤1.5; grade 2, >1.5–≤3; grade 3, >3–≤4.5; and grade 4, >4.5. Fig. 2 illustrates an example image corresponding to each rKOA grade. Additionally, as a sensitivity analysis, we adjusted mJSW measurements by normalizing them against the mean height of the population before assigning JSN grades (Supplementary Data S1, available at *Rheumatology Advances in Practice* online).

### Clinical outcomes

A binary variable indicating knee pain lasting for >3 months was created based on responses obtained from a questionnaire administered during the participants’ DXA appointment. Hospital-diagnosed KOA, hereafter referred to as HES-KOA, was determined using International Classification of Diseases (ICD) codes (9th and 10th revisions), which were obtained via linkage to Hospital Episodes Statistics (HES). Records began in 1997 and data were downloaded in July 2023, capturing information through the end of October 2022. This variable was analysed cross-sectionally, recognizing that KOA is a chronic condition that could have been present before the diagnosis. TKR had to be subsequent to the scan date and was based on Office of Population Censuses and Surveys (OPCS) codes, for which an associated date was obtained. None of the three clinical outcomes were side specific.

### Statistical analysis

Logistic regression was employed to investigate the associations between osteophytes, JSN and rKOA grades with knee pain and HES-KOA. The findings are presented as odds ratios (ORs) alongside their corresponding 95% CIs. When assessing the association of these exposures with TKR, Cox proportional hazards modelling was used, with results reported as hazard ratios (HRs) along with their 95% CIs. The proportional hazards assumption was checked using Schoenfeld residuals. Each exposure was compared against a reference group of individuals with a grade of 0 for that specific exposure. Both crude and adjusted models were conducted, with adjustments made for age, sex, height, weight and ethnicity (Supplementary Data S1, available at *Rheumatology Advances in Practice* online). The primary analysis included both males and females, with additional separate analyses conducted for each sex. Additionally, an interaction term for sex was incorporated into the primary models. All analyses were conducted using Stata version 17 (StataCorp, College Station, TX, USA).

## Results

### Population characteristics

In total, 19 595 right knee DXA scans were available after applying quality control measures. The mean age of participants was 63.7 years (range 45–82), with approximately equal sex distribution (51.8% females) (Table 1). A total of 2886 (14.7%) reported having had knee pain for >3 months, 917 (4.7%) had HES-KOA and 271 (1.4%) had undergone TKR after their DXA scan. The median time to TKR was 2.6 years (interquartile range 1.3–4.1).

**Table 1.** Baseline descriptive statistics of the study population

	All (*N* = 19 595)	Female (*n* = 10 146)	Male (*n* = 9449)
Demographics, mean (range)			
Age, years	63.73 (45–82)	63.03 (45–82)	64.49 (45–82)
Height, cm	170.20 (135–202)	163.59 (135–198)	177.29 (150–202)
Weight, kg	75.27 (36–169)	67.96 (36–169)	83.12 (48–160)
Ethnic background *n* (%)			
White	18 963 (96.77)	9827 (96.86)	9136 (96.69)
Asian	209 (1.07)	88 (0.87)	121 (1.28)
Chinese	54 (0.28)	36 (0.35)	18 (0.19)
Black	116 (0.59)	63 (0.62)	53 (0.56)
Mixed	91 (0.46)	49 (0.48)	42 (0.44)
Other	110 (0.56)	61 (0.60)	49 (0.52)
Unknown	52 (0.27)	22 (0.22)	30 (0.32)
Radiographic measures, *n* (%)			
OP any location	2359 (12.04)	1473 (14.52)	886 (9.38)
OP all locations	71 (0.36)	43 (0.42)	28 (0.30)
Medial femoral OP	1328 (6.78)	900 (8.87)	428 (4.53)
Lateral femoral OP	303 (1.55)	194 (1.91)	109 (1.15)
Medial tibial OP	1003 (5.12)	596 (5.87)	407 (4.31)
Lateral tibial OP	1243 (6.34)	748 (7.37)	495 (5.24)
Medial JSN	1847 (9.43)	1427 (14.06)	420 (4.44)
Radiographic measures, mean (range)			
Total OP area, mm^2^	24.28 (1.99–316.62)	22.88 (1.99–316.62)	26.59 (2.12–266.69)
Medial femoral OP area, mm^2^	18.29 (1.99–142.71)	17.28 (1.99–142.71)	20.42 (3.95–123.22)
Lateral femoral OP area, mm^2^	20.72 (2.46–155.11)	17.26 (2.46–68.56)	26.90 (3.29–155.11)
Medial tibial OP area, mm^2^	11.57 (2.05–118.09)	10.56 (2.05–118.09)	13.06 (2.12–95.09)
Lateral tibial OP area, mm^2^	12.14 (2.18–121.00)	11.38 (2.19–121.00)	13.28 (2.18–90.43)
Medial mJSW, mm	3.90 (0–7.27)	3.61 (0–6.31)	4.21 (0.37–7.27)
Lateral mJSW, mm	4.15 (0.47–7.87)	3.73 (0.48–7.74)	4.59 (0.47–7.87)
Clinical outcomes, *n* (%)			
Knee pain >3 months	2886 (14.73)	1500 (14.78)	1386 (14.67)
HES-KOA	917 (4.68)	425 (4.19)	492 (5.21)
TKR	271 (1.38)	141 (1.39)	130 (1.38)
Clinical outcomes, median (IQR)			
Time to TKR, years	2.60 (1.31–4.10)	2.54 (1.46–3.96)	2.68 (1.31–4.62)

IQR: interquartile range.

### Prevalence of rKOA

#### Osteophytes and JSN

Osteophytes (grade ≥1) were detected in 2359 (12.0%) DXA scans (Table 1), with the greatest prevalence observed in the medial femur, followed by the lateral tibia, medial tibia and lateral femur. Notably, females exhibited a higher frequency of osteophytes across all sites compared with males. Osteophytes tended to be larger on the femur than on the tibia. Medial JSN (grade ≥1) was present in 1847 participants (9.4%) and was almost three times more common in females. The prevalence of individual osteophyte and JSN grades can be found in Supplementary Tables S2 and Table S3, available at *Rheumatology Advances in Practice* online, respectively.

#### Overall rKOA grade

A classifier for rKOA was constructed by combining scores for osteophytes and JSN. The distribution of participants across different rKOA grades is detailed in Supplementary Table S4, available at *Rheumatology Advances in Practice* online, with participant characteristics categorized by rKOA grade presented in Supplementary Table S5, available at *Rheumatology Advances in Practice* online. Among the participants, 15 768 (80.5%) exhibited grade 0 rKOA, while 2883 (14.7%) had grade 1, 712 (3.6%) had grade 2, 158 (0.8%) had grade 3 and 74 (0.4%) had grade 4. Due to the small number of participants with KOA grade 4, rKOA grades 3 and 4 were combined in subsequent analyses. Among females, 26.1% had an rKOA grade ≥1, compared with 12.5% for males.

### Associations between rKOA and KOA outcomes

#### Osteophytes vs KOA outcomes

In adjusted analyses, the presence of one or more osteophytes (grade ≥1) at any site was associated with knee pain, HES-KOA and TKR, with progressively higher effect estimates [OR 3.38 (95% CI 3.06, 3.75), 4.67 (4.03, 5.42) and 7.51 (5.84, 9.66), respectively] (Table 2). Results from the unadjusted analysis, provided in Supplementary Table S6, available at *Rheumatology Advances in Practice* online, demonstrated similar associations, albeit with larger effect sizes. Osteophytes located at each knee region were related to all clinical outcomes, with lateral femoral and medial tibial osteophytes showing the strongest associations with HES-KOA and TKR. Results of the sex-stratified analysis are detailed in Supplementary Tables S7 and S8, available at *Rheumatology Advances in Practice* online. The point estimates for the associations of individual osteophyte sites (grade ≥1) with pain and TKR were higher in males than in females after adjustment. However, no evidence of a sex interaction was observed when an interaction term was included in the main model.

**Table 2.** The adjusted associations of osteophytes and JSN (grades ≥1) with KOA outcomes.

Association	Pain			HES-KOA			TKR		
	OR	95% CI	*P*-value	OR	95% CI	*P*-value	HR	95% CI	*P*-value
Any OP	3.38	3.74, 0.00	1.0 × 10^−123^	4.67	4.03, 5.42	2.7 × 10^−93^	7.51	5.84, 9.66	8.4 × 10^−56^
OP at all locations	3.90	6.30, 0.00	3.1 × 10^−8^	4.37	2.48, 7.68	3.2 × 10^−7^	5.77	2.94, 11.31	3.4 × 10^−7^
Medial femoral OP	3.50	3.96, 0.00	1.2 × 10^−87^	3.84	3.23, 4.57	8.5 × 10^−52^	5.20	3.98, 6.80	2.6 × 10^−33^
Lateral femoral OP	3.39	4.30, 0.00	1.7 × 10^−23^	6.19	4.71, 8.14	4.1 × 10^−39^	7.93	5.60, 11.23	2.0 × 10^−31^
Medial tibial OP	4.17	4.77, 0.00	2.8 × 10^−93^	5.43	4.55, 6.47	9.4 × 10^−39^	8.06	6.21, 10.46	1.9 × 10^−55^
Lateral tibial OP	2.90	3.30, 0.00	3.0 × 10^−59^	3.65	3.06, 4.36	6.1 × 10^−47^	4.86	3.72, 6.35	5.8 × 10^−31^
JSN	1.45	1.65, 0.00	7.0 × 10^−9^^a^	2.23	1.85, 2.67	1.0 × 10^−17^^a^	3.23	2.45, 4.26	8.0 × 10^−17^^a^

OP: osteophyte.

Logistic regression and Cox proportional hazards modelling results showing the associations of osteophyte and JSN grades (grade ≥1 *vs* 0) with knee pain, HES-KOA and TKR, respectively (*n* = 19 595). Models were adjusted for age, sex, height, weight and ethnicity.

a Denotes a sex interaction term with *P* < 0.05.

**Figure 3.**
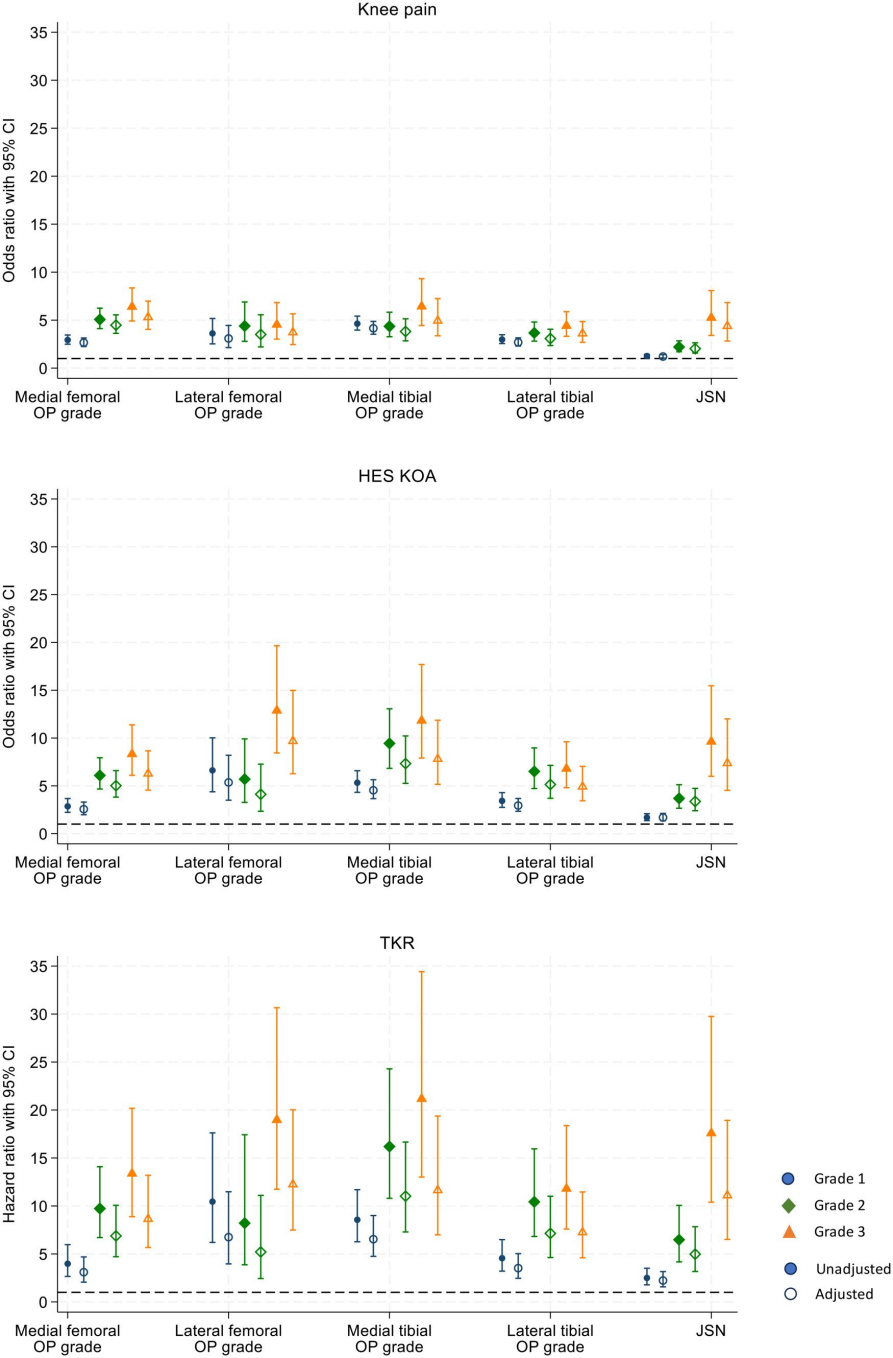
Associations of osteophyte grades and medial JSN grades with KOA outcomes. The graphs depict both crude and adjusted ORs and HRs, accompanied by 95% CIs, for knee pain, HES-KOA and TKR, across different grades of osteophytes and JSN (*n* = 19 595). Models are adjusted for age, sex, height, weight, and ethnicity. CI: Confidence Interval; HES-KOA: Knee Osteoarthritis based on Hospital Episode Statistics; JSN: Joint Space Narrowing; OP: Osteophyte; TKR: Total Knee Replacement

Higher osteophyte grades were generally more strongly associated with the three clinical outcomes at all four sites, in both unadjusted and adjusted analyses [Fig. 3; tabulated in Supplementary Tables S9, available at *Rheumatology Advances in Practice* online (unadjusted) and S10 (adjusted)]. However, some exceptions were observed: grade 2 medial tibial osteophytes showed slightly weaker associations with pain compared with grade 1 osteophytes; grade 3 lateral tibial osteophytes showed slightly weaker associations with HES-KOA compared with grade 2 osteophytes; grade 2 lateral femoral osteophytes showed slightly weaker associations with TKR compared with grade 1 osteophytes. Although the trend was less evident in the sex-stratified analyses (Supplementary Tables S11–S14, available at *Rheumatology Advances in Practice* online), the ORs and HRs for grade 3 osteophytes were generally larger than for grade 1. There was no evidence that sex modified the associations of osteophyte grades with clinical outcomes (Supplementary Tables S9 and S10, available at *Rheumatology Advances in Practice* online).

#### JSN vs KOA outcomes

JSN (grade ≥1) was associated with all three clinical outcomes, with effect sizes almost 50% less than that of osteophytes [knee pain: adjusted OR 1.45 (95% CI 1.28,1.65); HES-KOA: OR 2.23 (1.85, 2.67) and TKR: HR 3.23 (2.45, 4.26)] (Table 2). In sex-stratified analyses, ORs and HRs for the association of JSN (grade ≥1) with clinical outcomes were found to be higher in males compared with females (Supplementary Tables S7 and S8, available at *Rheumatology Advances in Practice* online) and there was evidence of a sex interaction for all outcomes (Table 2). As JSN grades increased, there was a corresponding increase in effect estimates (Fig. 3), which stronger associations for HES-KOA compared with pain and for TKR compared with HES-KOA. This trend remained consistent among females (Supplementary Tables S13 and S14, available at *Rheumatology Advances in Practice* online) but was less evident in males (Supplementary Tables S11 and S12, available at *Rheumatology Advances in Practice* online), although individual grades were still strongly associated with all three KOA outcomes. There was some evidence of a sex interaction [9, 10].

#### rKOA vs KOA outcomes

**Figure 4.**
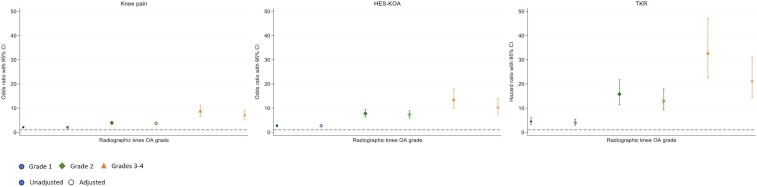
Associations of rKOA grade with knee osteoarthritis outcomes. The plots present both unadjusted and adjusted associations between rKOA grade, derived from a composite measure of osteophyte and JSN grades, and knee osteoarthritis outcomes (*n* = 19 595). The models include adjustments for age, sex, height, weight and ethnicity, with 95% CIs provided

The relationship between rKOA grades and clinical outcomes showed a consistent pattern of increasing strength of association with greater rKOA grade, which was observed across all three outcomes (Fig. 4; tabulated in Supplementary Table S15, available at *Rheumatology Advances in Practice* online). Adjusted ORs for pain ranged from 2.04 (95% CI 1.84, 2.26) for grade 1 rKOA to 7.08 (95% CI 5.41, 9.27) for grades 3–4. Similarly, for HES-OA, the adjusted ORs ranged from 2.67 (95% CI 2.25, 3.16) for grade 1 rKOA to 10.24 (95% CI 7.53, 13.93) for grades 3–4. Regarding TKR, HRs ranged from 3.97 (95% CI 2.90, 5.42) for grade 1 to 21.11 (95% CI 14.28, 31.19) for grades 3–4. In sex-stratified analyses (Supplementary Table S15, available at *Rheumatology Advances in Practice* online), rKOA measures remained associated with all three outcomes, with a clear progressive trend in females. In males, grade 2 rKOA had a stronger association with HES-KOA and TKR compared with grades 3–4, although grades 3–4 were still associated. Sex interactions were apparent for certain rKOA grades. Specifically, in adjusted models, there was evidence suggesting that the associations of rKOA grade 2 and rKOA grades 3–4 with HES-KOA and TKR were modified by sex. We conducted a sensitivity analysis to ensure that the observed associations between rKOA grades and clinical outcomes were not confounded by variations in participant height, as the shorter stature in females may have explained their greater prevalence of JSN. After deriving rKOA grades using height-normalized mJSW (prevalence detailed in Supplementary Table S16, available at *Rheumatology Advances in Practice* online), the results showed a similar sex interaction in the relationship between rKOA grade and clinical outcomes (Supplementary Table S17, available at *Rheumatology Advances in Practice* online).

## Discussion

We aimed to develop a novel classifier for rKOA based on knee DXA scans. To minimize subjective interpretation, we applied semi-automated techniques to annotate and grade osteophytes and JSN on scans from nearly 20 000 UKB participants to derive rKOA grades. Our study revealed an rKOA prevalence of 19.5%, consistent with previous estimates of rKOA based on X-rays, although reported ranges vary widely [22]. These variations likely stem from differences in participant selection, demographics and study characteristics. For instance, Cui et al. [23] reported prevalence rates of rKOA (defined as KL grade ≥2) ranging from 9 to 55% across 19 studies conducted between 2001 to 2020, with a pooled estimate of 28.7%. These studies were notably smaller in scale than the present investigation. Specifically, the study reporting a 9% prevalence rate included 1128 individuals from the USA with a mean age of 62 years (range 34–90), whereas the study with a 55% prevalence rate involved 3040 Japanese participants with a mean age of 70 years (s.d. 11). Our finding that women exhibited a higher rate than men (26.1% *vs* 12.5%) aligns with the existing literature, which consistently shows a higher prevalence of KOA among women, especially over the age of 40 years [24, 25].

To evaluate the face validity of our measure, we investigated associations with clinical outcomes related to KOA, namely prolonged knee pain, HES-KOA and subsequent TKR. These outcomes serve as proxies for increasing severity, with TKR representing end-stage OA. We observed robust and progressively increasing associations between grades of rKOA and all three outcomes. Furthermore, rKOA grades demonstrated stronger relationships with more advanced outcomes, with 7-fold, 10-fold and 19-fold increased risks of knee pain, HES-KOA and TKR for rKOA grades 3–4 compared with grade 0. These relationships appeared to reflect associations of both osteophyte and JSN grade with clinical outcomes, both of which were used to derive rKOA grades. That being said, pain correlated more strongly with the presence of osteophytes than with JSN, which is consistent with some studies [26, 27], but not all [28]. Taken together, these findings suggest that rKOA may have clinical relevance, given its relationship with outcomes such as pain and risk of TKR. While NICE guidelines prioritize a symptom-based diagnosis [29], our results suggest that imaging could be beneficial in certain cases, potentially complementing clinical assessments and aiding in treatment decisions.

Interestingly, we found evidence of a sex difference in the associations of JSN with KOA outcomes, as well as rKOA with pain, with generally higher effect estimates observed in males. Furthermore, the association between rKOA grade and HES-KOA and TKR was stronger in males at lower grades. This discrepancy may be attributed to narrower joint space width in healthy females compared with males, possibly due to their smaller stature, making JSN (derived from mJSW) a less specific measure for OA in females. This could in turn result in weaker associations with clinical outcomes. However, results of the sensitivity analysis show that these differences persist even after normalizing mJSW by mean height, suggesting that radiographic evidence of KOA may not correspond as closely with clinical outcomes in women. Other studies support this by demonstrating that, given the same level of radiographic severity, women tend to experience more intense pain and physical limitations than men [25, 30].

To our knowledge, no other automated or semi-automated rKOA classification system using knee DXA images has been reported. However, various machine learning and deep-learning methods have been applied to X-rays. Unlike our approach, which uses thresholds for JSN and osteophyte size, X-ray-based studies have typically trained models based on diagnoses made by radiologists. For example, Thomas et al. [10] developed an automated model using a convolutional neural network to detect rKOA (KL grade ≥ 2) from X-rays graded by radiologists, achieving performance comparable to human assessment. Similarly, Tiulpin et al. [31] employed deep-learning methods to accurately predict KL grade from knee X-rays, achieving an area under the ROC curve of 0.98 for detecting rKOA (KL grade ≥2).

Similar to the conventional KL grading system, our DXA-derived classification system prioritizes the assessment of osteophytes and JSN, as these features are considered hallmark signs of OA progression and have been associated with knee symptoms [32–36]. While sclerosis is a component of the KL grading system, definitive sclerosis was observed too infrequently on DXA images for inclusion in our classifier. This may represent a significant limitation given that some studies suggest sclerosis is associated with knee pain [28, 37]. On the other hand, our classifier offers several distinct advantages. For example, KL grading often introduces ambiguity, with terms like ‘definite’ osteophyte and ‘possible’ JSN (assuming a continuous progression of these structural changes), that can lead to discrepancies between raters and across studies [5–9]. In contrast, we automated the measurement of mJSW, enabling us to establish quantitative cut-offs for JSN. While osteophytes were manually identified, our classifier uses specific area-based cut-offs to define osteophyte grade, thereby avoiding the subjective scoring used in KL grading. Importantly, we observed strong correlations between these osteophyte grades and all three KOA outcomes, validating their use in future studies. By leveraging advancements in computer vision technologies, it may become feasible to fully automate the shading of osteophytes, enabling the widespread application of our classifier in large-scale epidemiological studies.

Another key strength of our method for evaluating rKOA is the lower radiation exposure of DXA scans compared with X-rays, making it particularly well-suited to repeated assessments over time. This approach could facilitate the evaluation of KOA-related structural changes in large cohorts, potentially uncovering new risk factors, including genetic ones, and leading to the identification of novel therapeutic targets. Additionally, since DXA scans are routinely used in osteoporosis screening, they offer an opportunity for simultaneous rKOA assessment, particularly in asymptomatic individuals at risk. This integration could facilitate earlier detection and intervention, improving outcomes and slowing disease progression.

In terms of limitations, although our clinical outcomes related to KOA were not side specific, our classification of rKOA was based solely on right knees. However, this approach likely reduces effect estimates rather than introducing biased associations. Additionally, HES-KOA, while specific, may be insensitive since obtaining an ICD code necessitates a hospital admission. Furthermore, since our classification system was developed using data from the UKB cohort, a predominantly Caucasian population, future studies should replicate our findings in diverse demographic groups to validate their broader applicability. A further limitation is that, unlike previous studies based on X-rays, DXA images are acquired with participants in a supine position, as opposed to weight bearing, meaning the mJSW is usually larger [38]. Like X-rays, being two-dimensional, DXA scans provide a limited view of osteophytes and can be distorted by minor changes in patient positioning, potentially obscuring osteophytes from view. The lower prevalence of lateral femoral osteophytes observed in our analysis compared with other sites may indicate potential issues related to rotation during image acquisition.

In conclusion, we have developed a semi-automated classifier for rKOA for use on knee DXA images, based on combinations of osteophytes at four locations within the knee joint and medial JSN. Having applied this classifier to right knee DXA images from ≈20 000 UKB participants, we observed expected prevalence rates for rKOA, including higher rates in females than males. Moreover, rKOA showed expected progressive associations with clinical outcomes, namely knee pain, HES-OA and TKR. Based on these findings, we propose that knee DXA scans could provide a valuable tool for ascertaining rKOA in large cohort studies, as well as pointing to their possible use in population-based screening.

## Supplementary Material

rkaf009_Supplementary_Data

## Data Availability

The data from this study will be available from UK Biobank in an upcoming data release. To access these resources, users must register with UK Biobank at: https://www.ukbiobank.ac.uk/enable-your-research/register. The BoneFinder^®^ knee module and markup tool are freely available upon request: https://bone-finder.com/.

## References

[1] Versus Arthritis. The state of musculoskeletal health 2023. Arthritis and other musculoskeletal conditions in numbers. 2023. https://www.versusarthritis.org/about-arthritis/data-and-statistics/the-state-of-musculoskeletal-health/ (10 October 2024, date last accessed).

[2] National Joint Registry. 20th annual report. 2023. https://reports.njrcentre.org.uk/Portals/0/PDFdownloads/NJR%2020th%20Annual%20Report%202023.pdf (10 October 2024, date last accessed).

[3] Matharu GS , Culliford DJ, Blom AW, Judge A. Projections for primary hip and knee replacement surgery up to the year 2060: an analysis based on data from the National Joint Registry for England, Wales, Northern Ireland and the Isle of Man. Ann R Coll Surg Engl 2022;104:443–8. 34939832 10.1308/rcsann.2021.0206 PMC9157920

[4] Kellgren JH , Lawrence JS. Radiological assessment of osteo-arthrosis. Ann Rheum Dis 1957;16:494–502. 13498604 10.1136/ard.16.4.494 PMC1006995

[5] Kohn MD , Sassoon AA, Fernando ND. Classifications in brief: Kellgren-Lawrence classification of osteoarthritis. Clin Orthop Relat Res 2016;474:1886–93. 26872913 10.1007/s11999-016-4732-4 PMC4925407

[6] Riddle DL , Jiranek WA, Hull JR. Validity and reliability of radiographic knee osteoarthritis measures by arthroplasty surgeons. Orthopedics 2013;36:e25–32. 23276348 10.3928/01477447-20121217-14

[7] Wright RW , Group M. Osteoarthritis classification scales: interobserver reliability and arthroscopic correlation. J Bone Joint Surg Am 2014;96:1145–51. 25031368 10.2106/JBJS.M.00929 PMC4083772

[8] Kessler S , Guenther KP, Puhl W. Scoring prevalence and severity in gonarthritis: the suitability of the Kellgren & Lawrence scale. Clin Rheumatol 1998;17:205–9. 9694053 10.1007/BF01451048

[9] Sheehy L , Culham E, McLean L et al Validity and sensitivity to change of three scales for the radiographic assessment of knee osteoarthritis using images from the Multicenter Osteoarthritis Study (MOST). Osteoarthritis Cartilage 2015;23:1491–8. 26003948 10.1016/j.joca.2015.05.003 PMC4831715

[10] Thomas KA , Kidziński Ł, Halilaj E et al Automated classification of radiographic knee osteoarthritis severity using deep neural networks. Radiol Artif Intell 2020;2:e190065. 32280948 10.1148/ryai.2020190065 PMC7104788

[11] Tiulpin A , Thevenot J, Rahtu E, Lehenkari P, Saarakkala S. Automatic knee osteoarthritis diagnosis from plain radiographs: a deep learning-based approach. Sci Rep 2018;8:1727. 29379060 10.1038/s41598-018-20132-7 PMC5789045

[12] Pi SW , Lee BD, Lee MS, Lee HJ. Ensemble deep-learning networks for automated osteoarthritis grading in knee X-ray images. Sci Rep 2023;13:22887. 38129653 10.1038/s41598-023-50210-4 PMC10739741

[13] Yoon JS , Yon C-J, Lee D et al Assessment of a novel deep learning-based software developed for automatic feature extraction and grading of radiographic knee osteoarthritis. BMC Musculoskelet Disord 2023;24:869. 37940935 10.1186/s12891-023-06951-4 PMC10631128

[14] Swiecicki A , Li N, O’Donnell J et al Deep learning-based algorithm for assessment of knee osteoarthritis severity in radiographs matches performance of radiologists. Comput Biol Med 2021;133:104334. 33823398 10.1016/j.compbiomed.2021.104334

[15] Oka H , Muraki S, Akune T et al Fully automatic quantification of knee osteoarthritis severity on plain radiographs. Osteoarthritis Cartilage 2008;16:1300–6. 18424107 10.1016/j.joca.2008.03.011

[16] Yoshida K , Barr RJ, Galea-Soler S et al Reproducibility and diagnostic accuracy of Kellgren-Lawrence grading for osteoarthritis using radiographs and dual-energy X-ray absorptiometry images. J Clin Densitom 2015;18:239–44. 25304911 10.1016/j.jocd.2014.08.003

[17] Damilakis J , Adams JE, Guglielmi G, Link TM. Radiation exposure in X-ray-based imaging techniques used in osteoporosis. Eur Radiol 2010;20:2707–14. 20559834 10.1007/s00330-010-1845-0 PMC2948153

[18] Littlejohns TJ , Holliday J, Gibson LM et al The UK Biobank imaging enhancement of 100,000 participants: rationale, data collection, management and future directions. Nat Commun 2020;11:2624. 32457287 10.1038/s41467-020-15948-9 PMC7250878

[19] Faber BG , Ebsim R, Saunders FR et al A novel semi-automated classifier of hip osteoarthritis on DXA images shows expected relationships with clinical outcomes in UK Biobank. Rheumatology (Oxford) 2022;61:3586–95. 34919677 10.1093/rheumatology/keab927 PMC9434243

[20] Beynon RA , Saunders FR, Ebsim R et al Dual-energy X-ray absorptiometry derived knee shape may provide a useful imaging biomarker for predicting total knee replacement: findings from a study of 37,843 people in UK Biobank. Osteoarthr Cartil Open 2024;6:100468. 38655015 10.1016/j.ocarto.2024.100468 PMC11035060

[21] Lindner C , Thiagarajah S, Wilkinson JM et al Fully automatic segmentation of the proximal femur using random forest regression voting. IEEE Trans Med Imaging 2013;32:1462–72. 23591481 10.1109/TMI.2013.2258030

[22] Pereira D , Peleteiro B, Araujo J et al The effect of osteoarthritis definition on prevalence and incidence estimates: a systematic review. Osteoarthritis Cartilage 2011;19:1270–85. 21907813 10.1016/j.joca.2011.08.009

[23] Cui A , Li H, Wang D et al Global, regional prevalence, incidence and risk factors of knee osteoarthritis in population-based studies. EClinicalMedicine 2020;29-30:100587. 34505846 10.1016/j.eclinm.2020.100587 PMC7704420

[24] Srikanth VK , Fryer JL, Zhai G et al A meta-analysis of sex differences prevalence, incidence and severity of osteoarthritis. Osteoarthritis Cartilage 2005;13:769–81. 15978850 10.1016/j.joca.2005.04.014

[25] Segal NA , Nilges JM, Oo WM. Sex differences in osteoarthritis prevalence, pain perception, physical function and therapeutics. Osteoarthritis Cartilage 2024;32:1045–53. 38588890 10.1016/j.joca.2024.04.002

[26] Lanyon P , O’Reilly S, Jones A, Doherty M. Radiographic assessment of symptomatic knee osteoarthritis in the community: definitions and normal joint space. Ann Rheum Dis 1998;57:595–601. 9893570 10.1136/ard.57.10.595 PMC1752476

[27] Cicuttini FM , Baker J, Hart DJ, Spector TD. Association of pain with radiological changes in different compartments and views of the knee joint. Osteoarthritis Cartilage 1996;4:143–7. 8806116 10.1016/s1063-4584(05)80323-1

[28] Neogi T , Felson D, Niu J et al Association between radiographic features of knee osteoarthritis and pain: results from two cohort studies. BMJ 2009;339:b2844. 19700505 10.1136/bmj.b2844 PMC2730438

[29] National Institute for Health and Care Excellence. Osteoarthritis in over 16s: diagnosis and management. 2022. https://www.nice.org.uk/guidance/ng226/chapter/Recommendations#diagnosis (17 July 2024, date last accessed). 36745715

[30] Tschon M , Contartese D, Pagani S, Borsari V, Fini M. Gender and sex are key determinants in osteoarthritis not only confounding variables. A systematic review of clinical data. J Clin Med 2021;10:3178. 34300344 10.3390/jcm10143178 PMC8303951

[31] Tiulpin A , Saarakkala S. Automatic grading of individual knee osteoarthritis features in plain radiographs using deep convolutional neural networks. Diagnostics (Basel) 2020;10:932. 33182830 10.3390/diagnostics10110932 PMC7697270

[32] Lethbridge-Cejku M , Scott WW, Reichle R et al Association of radiographic features of osteoarthritis of the knee with knee pain: data from the Baltimore Longitudinal Study of Aging. Arthritis Care Res 1995;8:182–8. 7654803 10.1002/art.1790080311

[33] Fan T , Ruan G, Antony B et al The interactions between MRI-detected osteophytes and bone marrow lesions or effusion-synovitis on knee symptom progression: an exploratory study. Osteoarthritis Cartilage 2021;29:1296–305. 34216729 10.1016/j.joca.2021.06.008

[34] Kornaat PR , Bloem JL, Ceulemans RYT et al Osteoarthritis of the knee: association between clinical features and MR imaging findings. Radiology 2006;239:811–7. 16714463 10.1148/radiol.2393050253

[35] Muraki S , Akune T, En-Yo Y et al Joint space narrowing, body mass index, and knee pain: the ROAD study (OAC1839R1). Osteoarthritis Cartilage 2015;23:874–81. 25639569 10.1016/j.joca.2015.01.011

[36] Muraki S , Oka H, Akune T et al Independent association of joint space narrowing and osteophyte formation at the knee with health-related quality of life in Japan: a cross-sectional study. Arthritis Rheum 2011;63:3859–64. 21898346 10.1002/art.30641

[37] Szebenyi B , Hollander AP, Dieppe P et al Associations between pain, function, and radiographic features in osteoarthritis of the knee. Arthritis Rheum 2006;54:230–5. 16385522 10.1002/art.21534

[38] Abdullah SS , Rajasekaran MP. Do weight-bearing knee digital radiographs help to track the severity of OA? Indian J Orthop 2022;56:664–71. 35342524 10.1007/s43465-021-00560-w PMC8921390

