# Supplementary material for: A novel classifier of radiographic knee osteoarthritis for use on knee DXA images is predictive of joint replacement in UK Biobank
Source: Rheumatol Adv Pract. 2025 Jan 20;9(1):rkaf009. doi: 10.1093/rap/rkaf009 (PMC11846665; doi:10.1093/rap/rkaf009)
Supplement: rkaf009_Supplementary_Data [file rkaf009_supplementary_data.zip › 1d814_24-234 Supplementary Material.docx]

# Supplementary Tables

| **Combined score** | **N** | **%** |
| --- | --- | --- |
| 0 | 15,768 | 80.47 |
| 0.5 | 823 | 4.2 |
| 1 | 1,678 | 8.56 |
| 1.5 | 382 | 1.95 |
| 2 | 428 | 2.18 |
| 2.5 | 142 | 0.72 |
| 3 | 142 | 0.72 |
| 3.5 | 71 | 0.36 |
| 4 | 55 | 0.28 |
| 4.5 | 32 | 0.16 |
| 5 | 29 | 0.15 |
| 5.5 | 17 | 0.09 |
| 6 | 11 | 0.06 |
| 6.5 | 7 | 0.04 |
| 7 | 3 | 0.02 |
| 7.5 | 5 | 0.03 |
| 8 | 1 | 0.01 |
| 9 | 1 | 0.01 |

Supplementary Table S1: The breakdown of the combine score including osteophyte and JSN grades.

Radiographic osteophyte grades, initially multiplied by 0.5, were summed across the four sites on the medial and lateral aspects of the femur and tibia, with a maximum possible score of 6. This score was then combined with the JSN grade, resulting in a maximum possible score of 9 for each individual. Cut-offs were used to assign overall rKOA grades based on these combined scores.

|  | **All**  (n=19,595) | | **Female**  (n=10,146) | | **Male**  (n=9,449) | |
| --- | --- | --- | --- | --- | --- | --- |
| **Osteophyte grade** | N | % | N | % | N | % |
| Medial Femur |  |  |  |  |  |  |
| 0 | 18,267 | (93.22) | 9,246 | (91.13) | 9,021 | (95.47) |
| 1 | 732 | (3.74) | 535 | (5.27) | 197 | (2.08) |
| 2 | 374 | (1.91) | 234 | (2.31) | 140 | (1.48) |
| 3 | 222 | (1.13) | 131 | (1.29) | 91 | (0.96) |
| Lateral femur |  |  |  |  |  |  |
| 0 | 19,292 | (98.45) | 9,952 | (98.09) | 9,340 | (98.85) |
| 1 | 130 | (0.66) | 100 | (0.99) | 30 | (0.32) |
| 2 | 78 | (0.40) | 49 | (0.48) | 29 | (0.31) |
| 3 | 95 | (0.48) | 45 | (0.44) | 50 | (0.53) |
| Medial tibia |  |  |  |  |  |  |
| 0 | 18,592 | (94.88) | 9,550 | (94.13) | 9,042 | (95.69) |
| 1 | 695 | (3.55) | 453 | (4.46) | 242 | (2.56) |
| 2 | 195 | (1.00) | 90 | (0.89) | 105 | (1.11) |
| 3 | 113 | (0.58) | 53 | (0.52) | 60 | (0.63) |
| Lateral tibia |  |  |  |  |  |  |
| 0 | 18,352 | (93.66) | 9,398 | (92.63) | 8,954 | (94.76) |
| 1 | 809 | (4.13) | 502 | (4.95) | 307 | (3.25) |
| 2 | 237 | (1.21) | 141 | (1.39) | 96 | (1.02) |
| 3 | 197 | (1.01) | 105 | (1.03) | 92 | (0.97) |

Supplementary Table S2: Prevalence of Osteophytes by site.

|  | **All** (n=19,595) | **Female** (n=10,146) | **Male** (n=9,449) |
| --- | --- | --- | --- |
| **Medial JSN grade** |  |  |  |
| 0 | 17748 (90.57%) | 8719 (85.94%) | 9029 (95.56%) |
| 1 | 1448 (7.39%) | 1129 (11.13%) | 319 (3.38%) |
| 2 | 315 (1.61%) | 248 (2.44%) | 67 (0.71%) |
| 3 | 84 (0.43%) | 50 (0.49%) | 34 (0.36%) |

Supplementary Table S3: Prevalence of medial joint space narrowing (JSN).

|  | **All** (n=19,595) | **Female** (n=10,146) | **Male** (n=9,449) |
| --- | --- | --- | --- |
| **rKOA grade** |  |  |  |
| 0 | 15768 (80.47%) | 7500 (73.92%) | 8268 (87.50%) |
| 1 | 2883 (14.71%) | 2027 (19.98%) | 856 (9.06%) |
| 2 | 712 (3.63%) | 492 (4.85%) | 220 (2.33%) |
| 3 | 158 (0.81%) | 87 (0.86%) | 71 (0.75%) |
| 4 | 74 (0.38%) | 40 (0.39%) | 34 (0.36%) |

Supplementary Table S4: Prevalence of radiographic knee osteoarthritis (rKOA).

|  | **rKOA grade** | | | |
| --- | --- | --- | --- | --- |
|  | 1 | 2 | 3 | 4 |
|  | N=2883 | N=712 | N=158 | N=74 |
| **Demographics** | Mean (SD) | | | |
| Age (years) | 65.22 (7.00) | 66.44 (6.89) | 66.66 (6.38) | 66.76 (6.13) |
| Height (cm) | 167.27 (9.08) | 167.28 (9.48) | 169.84 (9.70) | 168.69 (9.99) |
| Weight (kg) | 74.24 (15.76) | 76.39 (16.63) | 82.61 (17.49) | 90.00 (21.88) |
| **Radiographic measures** | Frequency (%) | | | |
| OP at any site | 1668 (57.86%) | 459 (64.47%) | 158 (100.00%) | 74 (100.00%) |
| OP at all sites | 0 (0.00%) | 15 (2.11%) | 29 (18.35%) | 27 (36.49%) |
| Medial femoral OP | 805 (27.92%) | 326 (45.79%) | 130 (82.28%) | 67 (90.54%) |
| Lateral femoral OP | 89 (3.09%) | 114 (16.01%) | 66 (41.77%) | 34 (45.95%) |
| Medial tibial OP | 518 (17.97%) | 286 (40.17%) | 129 (81.65%) | 70 (94.59%) |
| Lateral tibial OP | 773 (26.81%) | 294 (41.29%) | 115 (72.78%) | 61 (82.43%) |
| Medial JSN | 1312 (45.51%) | 393 (55.20%) | 82 (51.90%) | 60 (81.08%) |
| **Clinical outcomes** | Frequency (%) | | | |
| Knee pain | 635 (22.03%) | 245 (34.41%) | 83 (52.53%) | 42 (56.76%) |
| HES-KOA | 225 (7.80%) | 140 (19.66%) | 45 (28.48%) | 24 (32.43%) |
| Knee replacement | 75 (2.60%) | 62 (8.71%) | 25 (15.82%) | 15 (20.27%) |

Supplementary Table S5: Participant characteristics by radiographic KOA grade.

Abbreviations: cm, centimetres; HES-KOA, hospital diagnosed knee osteoarthritis; JSN, joint space narrowing; Kg, kilograms; OP, osteophytes; TKR, total knee replacement.

|  | **Pain** | | |  | **HES-KOA** | | |  | **TKR** | | |
| --- | --- | --- | --- | --- | --- | --- | --- | --- | --- | --- | --- |
|  | OR | 95% CI | *p* |  | OR | 95% CI | *p* |  | HR | 95% CI | *p* |
| Any OP | 3.72 | (3.37, 4.10) | <1.0 x 10^-40^ |  | 5.39 | (4.69, 6.21) | <1.0 x 10^-40^ |  | 9.74 | (7.67, 12.38) | <1.0 x 10^-40^ |
| OP at all locations | 5.07 | (3.18, 8.10) | 1.04 x10^-11^ |  | 6.51 | (3.76, 11.28) | 2.26 x 10^-11^ |  | 10.52 | (5.41, 20.44) | 3.93 x 10^-12^ |
| Medial femoral OP | 3.95 | (3.50, 4.45) | <1.0 x 10^-40^ |  | 4.53 | (3.84, 5.35) | <1.0 x 10^-40^ |  | 7.10 | (5.50, 9.18) | <1.0 x 10^-40^ |
| Lateral femoral OP | 4.09 | (3.24, 5.16) | 2.04 x10^-32^ |  | 8.05 | (6.19, 10.49) | <1.0 x 10^-40^ |  | 12.36 | (8.83, 17.29) | <1.0 x 10^-40^ |
| Medial tibial OP | 4.75 | (4.16, 5.43) | <1.0 x 10^-40^ |  | 6.70 | (5.65, 7.94) | <1.0 x 10^-40^ |  | 11.32 | (8.82, 14.53) | <1.0 x 10^-40^ |
| Lateral tibial OP | 3.31 | (2.93, 3.76) | <1.0 x 10^-40^ |  | 4.48 | (3.77, 5.31) | <1.0 x 10^-40^ |  | 6.76 | (5.22, 8.75) | <1.0 x 10^-40^ |
| JSN | 1.53 | (1.35, 1.73) | 7.19 x 10^-12^* |  | 2.29 | (1.92, 2.72) | 1.26 x 10^-20^* |  | 3.80 | (2.92, 4.95) | 3.85 x 10^-23^* |

Supplementary Table S6: The unadjusted associations of osteophytes and joint space narrowing (grades >1) with knee OA outcomes in males and females combined.

Abbreviations: CI, Confidence Interval; HES-KOA, Knee osteoarthritis based on Hospital Episodes Statistics; HR, Hazard Ratio; JSN, Joint Space Narrowing; OP, Osteophyte; OR Odds Ratio; TKR, Total Knee Replacement. *Denotes a sex-interaction term with p<0.05. n=19,595.

|  | **Pain** | | |  | **HES-KOA** | | |  | **TKR** | | |
| --- | --- | --- | --- | --- | --- | --- | --- | --- | --- | --- | --- |
|  | *Unadjusted* | | | | | | | | | | |
|  | OR | 95% CI | *p* |  | OR | 95% CI | *p* |  | HR | 95% CI | *p* |
| Any OP | 3.72 | (3.20, 4.33) | <1.0 x 10^-40^ |  | 5.12 | (4.17, 6.28) | <1.0 x 10^-40^ |  | 10.45 | (7.41, 14.74) | <1.0 x 10^-40^ |
| OP at all locations | 5.87 | (2.79, 12.33) | 3.05 x 10^-06^ |  | 6.14 | (2.60, 14.52) | 3.54 x 10^-05^ |  | 11.06 | (4.09, 29.92) | 2.24 x 10^-06^ |
| Medial femoral OP | 4.41 | (3.61, 5.40) | <1.0 x 10^-40^ |  | 4.63 | (3.56, 6.04) | 6.45 x 10^-30^ |  | 8.68 | (5.88, 12.80) | 1.15 x 10^-27^ |
| Lateral femoral OP | 4.89 | (3.34, 7.16) | 3.76 x 10^-16^ |  | 8.02 | (5.26, 12.24) | 4.74 x 10^-22^ |  | 13.57 | (8.04, 22.90) | 1.68 x 10^-22^ |
| Medial tibial OP | 4.82 | (3.93, 5.93) | <1.0 x 10^-40^ |  | 6.42 | (4.99, 8.26) | <1.0 x 10^-40^ |  | 13.67 | (9.54, 19.60) | <1.0 x 10^-40^ |
| Lateral tibial OP | 3.23 | (2.66, 3.93) | 8.43 x 10^-32^ |  | 4.14 | (3.20, 5.35) | 2.30 x 10^-27^ |  | 6.86 | (4.65, 10.10) | 2.15 x 10^-22^ |
| JSN | 2.64 | (2.13, 3.28) | 1.69 x 10^-18^ |  | 3.77 | (2.85, 4.98) | 1.81 x 10^-20^ |  | 5.77 | (3.80, 8.77) | 2.17 x 10^-16^ |
|  | *Adjusted* | | | | | | | | | | |
| Any OP | 3.46 | (2.96, 4.04) | <1.0 x 10^-40^ |  | 4.40 | (3.57, 5.43) | <1.0 x 10^-40^ |  | 8.36 | (5.86, 11.91) | 7.64 x 10^-32^ |
| OP at all locations | 4.33 | (2.03, 9.23) | 1.52 x 10^-04^ |  | 4.03 | (1.68, 9.68) | 0.002 |  | 6.03 | (2.19, 16.59) | 5.00 x 10^-04^ |
| Medial femoral OP | 4.04 | (3.29, 4.96) | <1.0 x 10^-40^ |  | 3.86 | (2.95, 5.06) | 1.01 x 10^-22^ |  | 6.59 | (4.42, 9.82) | 2.11 x 10^-20^ |
| Lateral femoral OP | 4.23 | (2.87, 6.24) | 3.51 x 10^-13^ |  | 6.34 | (4.11, 9.76) | 5.45 x 10^-17^ |  | 9.20 | (5.38, 15.72) | 4.94 x 10^-16^ |
| Medial tibial OP | 4.36 | (3.53, 5.37) | <1.0 x 10^-40^ |  | 5.33 | (4.12, 6.91) | 7.63 x 10^-37^ |  | 10.48 | (7.21, 15.23) | 6.48 x 10^-35^ |
| Lateral tibial OP | 2.92 | (2.39, 3.56) | 8.07 x 10^-26^ |  | 3.45 | (2.66, 4.49) | 1.97 x 10^-20^ |  | 5.21 | (3.50, 7.75) | 4.03 x 10^-16^ |
| JSN | 2.50 | (2.00, 3.12) | 6.44 x 10^-16^ |  | 3.25 | (2.44, 4.33) | 7.60 x 10^-16^ |  | 4.45 | (2.90, 6.82) | 7.26 x 10^-12^ |

Supplementary Table 7: The associations of osteophytes and joint space narrowing (grades *>*1) with knee OA outcomes in males. Abbreviations: CI, Confidence Interval; HES-KOA, Knee osteoarthritis based on Hospital Episodes Statistics; HR, Hazard Ratio; JSN, Joint Space Narrowing; OP, Osteophyte; OR Odds Ratio; TKR, Total Knee Replacement. Models were adjusted for age, height, weight and ethnic group. n=9,449.

|  | **Pain** | | |  | **HES-KOA** | | |  | **TKR** | | |
| --- | --- | --- | --- | --- | --- | --- | --- | --- | --- | --- | --- |
|  | *Unadjusted* | | | | | | | | | | |
|  | OR | 95% CI | *p* |  | OR | 95% CI | *p* |  | HR | 95% CI | *p* |
| Any OP | 3.78 | (3.33, 4.29) | <1.0 x 10^-40^ |  | 6.28 | (5.14, 7.66) | <1.0 x 10^-40^ |  | 9.56 | (6.82, 13.39) | <1.0 x 10^-40^ |
| OP at all locations | 4.61 | (2.52, 8.43) | 7.23 x 10^-07^ |  | 7.07 | (3.46, 14.45) | 7.95 x10^-08^ |  | 10.12 | (4.14, 24.71) | 3.76 x 10^-07^ |
| Medial femoral OP | 3.79 | (3.27, 4.40) | <1.0 x 10^-40^ |  | 5.00 | (4.01, 6.22) | <1.0 x 10^-40^ |  | 6.42 | (4.55, 9.05) | 3.77 x 10_-26_ |
| Lateral femoral OP | 3.69 | (2.74, 4.95) | 4.44 x10^-18^ |  | 8.61 | (6.13, 12.09) | 2.21 x 10^-35^ |  | 11.78 | (7.59, 18.29) | 3.87 x 10^-28^ |
| Medial tibial OP | 4.72 | (3.97, 5.62) | <1.0 x 10^-40^ |  | 7.33 | (5.81, 9.24) | <1.0 x 10^-40^ |  | 9.69 | (6.86, 13.69) | 5.55 x 10^-38^ |
| Lateral tibial OP | 3.39 | (2.88, 3.98) | <1.0 x 10^-40^ |  | 5.04 | (4.00, 6.35) | <1.0 x 10^-40^ |  | 6.76 | (4.77, 9.57) | 6.14 x 10^-27^ |
| JSN | 1.24 | (1.07, 1.44) | 0.005 |  | 2.03 | (1.61, 2.55) | 1.48 x 10^-09^ |  | 3.26 | (2.30, 4.61) | 2.42 x 10^-11^ |
|  | *Adjusted* | | | | | | | | | | |
| Any OP | 3.32 | (2.90, 3.79) | <1.0 x 10^-40^ |  | 4.92 | (3.99, 6.07) | <1.0 x 10^-40^ |  | 6.75 | (4.74, 9.62) | 3.76 x 10^-26^ |
| OP at all locations | 3.65 | (1.95, 6.83) | 7.23 x 10^-07^ |  | 4.62 | (2.20, 9.70) | 7.95 x 10^-08^ |  | 5.45 | (2.20, 13.51) | 2.47 x 10^-04^ |
| Medial femoral OP | 3.21 | (2.75, 3.75) | <1.0 x 10^-40^ |  | 3.75 | (2.98, 4.72) | <1.0 x 10^-40^ |  | 4.30 | (3.00, 6.14) | 1.34 x 10^-15^ |
| Lateral femoral OP | 2.92 | (2.15, 3.97) | 4.44 x 10^-18^ |  | 6.01 | (4.21, 8.56) | 2.21 x 10^-35^ |  | 7.03 | (4.45, 11.10) | 6.94 x 10^-17^ |
| Medial tibial OP | 4.01 | (3.35, 4.80) | <1.0 x 10^-40^ |  | 5.45 | (4.28, 6.94) | <1.0 x 10^-40^ |  | 6.35 | (4.43, 9.11) | 1.01 x 10^-23^ |
| Lateral tibial OP | 2.88 | (2.43, 3.40) | <1.0 x 10^-40^ |  | 3.78 | (2.97, 4.81) | <1.0 x 10^-40^ |  | 4.53 | (3.16, 6.51) | 2.88 x 10^-16^ |
| JSN | 1.16 | (1.00, 1.35) | 0.005 |  | 1.77 | (1.40, 2.24) | 1.48 x 10^-09^ |  | 2.65 | (1.87, 3.77) | 5.60 x 10^-08^ |

Supplementary Table S8: The associations of osteophytes and joint space narrowing (grades >1) with knee OA outcomes in females. Abbreviations: CI, Confidence Interval; HES-KOA, Knee osteoarthritis Based on Hospital Episodes Statistics; HR, Hazard Ratio; JSN, Joint Space Narrowing; OP, Osteophyte; OR Odds Ratio; TKR, Total Knee Replacement. Models were adjusted for age, height, weight and ethnic group. n=10,146.

|  | **Pain** | | |  | **HES KOA** | | |  | **TKR** | | |
| --- | --- | --- | --- | --- | --- | --- | --- | --- | --- | --- | --- |
|  | OR | 95% CI | *p* |  | OR | 95% CI | *p* |  | HR | 95% CI | *p* |
| **Medial femur** |  |  |  |  |  |  |  |  |  |  |  |
| grade 1 | 2.93 | (2.49, 3.45) | 3.50 x 10^-38^ |  | 2.86 | (2.23, 3.67) | 1.32 x 10^-16^ |  | 3.98 | (2.66, 5.97) | 2.05 x 10^-11^ |
| grade 2 | 5.07 | (4.12, 6.25) | <1.0 x 10^-40^ |  | 6.09 | (4.67, 7.94) | <1.0 x 10^-40^ |  | 9.73 | (6.71, 14.09) | 2.76 x 10^-33^ |
| grade 3 | 6.40 | (4.91, 8.36) | <1.0 x 10^-40^ |  | 8.33 | (6.10, 11.38) | <1.0 x 10^-40^ |  | 13.38 | (8.88, 20.18) | 3.46 x 10^-35^ |
| **Lateral femur** |  |  |  |  |  |  |  |  |  |  |  |
| grade 1 | 3.62 | (2.53, 5.17) | 1.65 x 10^-12^ |  | 6.62 | (4.38, 10.02) | 3.44 x 10^-19^ |  | 10.45 | (6.20, 17.62) | 1.30 x 10^-18^ |
| grade 2 | 4.39 | (2.79, 6.89) | 1.31 x 10^-10^ |  | 5.70 | (3.27, 9.91) | 7.44 x 10^-10^ |  | 8.21 | (3.87, 17.42) | 4.07 x 10^-08^ |
| grade 3 | 4.54 | (3.02, 6.83) | 3.59 x 10^-13^ |  | 12.88 | (8.44, 19.65) | 2.09 x 10^-32^ |  | 18.97 | (11.74, 30.66) | 3.05 x 10^-33^ |
| **Medial tibia** |  |  |  |  |  |  |  |  |  |  |  |
| grade 1 | 4.63 | (3.96, 5.42) | <1.0 x 10^-40^ |  | 5.33 | (4.32, 6.58) | <1.0 x 10^-40^ |  | 8.56 | (6.27, 11.70) | <1.0 x 10^-40^ |
| grade 2 | 4.36 | (3.27, 5.83) | 2.05 x 10^-23^ |  | 9.44 | (6.83, 13.06) | <1.0 x 10^-40^ |  | 16.19 | (10.80, 24.29) | <1.0 x 10^-40^ |
| grade 3 | 6.43 | (4.44, 9.32) | 8.65 x 10^-23^ |  | 11.83 | (7.91, 17.69) | 2.77 x 10^-33^ |  | 21.17 | (13.01, 34.42) | 9.02 x 10^-35^ |
| **Lateral tibia** |  |  |  |  |  |  |  |  |  |  |  |
| grade 1 | 2.98 | (2.56, 3.48) | <1.0 x 10^-40^ |  | 3.43 | (2.75, 4.29) | 1.37 x 10^-27^ |  | 4.56 | (3.21, 6.49) | 3.24 x 10^-17^ |
| grade 2 | 3.67 | (2.81, 4.80) | 1.95 x 10^-21^ |  | 6.51 | (4.72, 8.97) | 2.45 x 10^-30^ |  | 10.43 | (6.82, 15.95) | 2.88 x 10^-27^ |
| grade 3 | 4.41 | (3.31, 5.88) | 4.76 x 10^-24^ |  | 6.80 | (4.81, 9.61) | 1.92 x 10^-27^ |  | 11.81 | (7.59, 18.37) | 6.24 x 10^-28^ |
| **JSN** |  |  |  |  |  |  |  |  |  |  |  |
| Grade 1 | 1.26 | (1.09, 1.45) | 0.002* |  | 1.69 | (1.36, 2.09) | 2.12 x 10^-06^* |  | 2.50 | (1.78, 3.51) | 1.37 x 10^-07^* |
| Grade 2 | 2.20 | (1.71, 2.84) | 9.71 x 10^-10^* |  | 3.69 | (2.66, 5.12) | 4.92 x 10^-15^* |  | 6.48 | (4.17, 10.06) | 1.01 x 10^-16^ |
| Grade 3 | 5.25 | (3.41, 8.08) | 4.54 x 10^-14^ |  | 9.63 | (6.00, 15.46) | 6.81 x 10^-21^* |  | 17.59 | (10.40, 29.75) | 1.05 x 10^-26^* |

Supplementary Table S9: Unadjusted associations of OP grades and JSN grades with knee OA outcomes in males and females combined.

Abbreviations: CI, Confidence Interval; HES-KOA, Knee osteoarthritis based on Hospital Episodes Statistics; HR, Hazard Ratio; JSN, Joint Space Narrowing; OP, Osteophyte; OR Odds Ratio; TKR, Total Knee Replacement. *Denotes a sex-interaction term with p<0.05. n=19,595.

|  | **Pain** | | |  | **HES KOA** | | |  | **TKR** | | |
| --- | --- | --- | --- | --- | --- | --- | --- | --- | --- | --- | --- |
|  | OR | 95% CI | *p* |  | OR | 95% CI | *p* |  | HR | 95% CI | *p* |
| **Medial femur** |  |  |  |  |  |  |  |  |  |  |  |
| grade 1 | 2.66 | (2.25, 3.14) | 2.14 x 10^-30^ |  | 2.56 | (1.98, 3.30) | 5.86 x 10^-13^ |  | 3.10 | (2.06, 4.69) | 7.08 x 10^-08^ |
| grade 2 | 4.48 | (3.62, 5.55) | <1.0 x 10^-40^ |  | 5.02 | (3.82, 6.59) | 4.15 x 10^-31^ |  | 6.88 | (4.71, 10.07) | 2.46 x 10^-23^ |
| grade 3 | 5.31 | (4.04, 6.98) | 3.76 x 10^-33^ |  | 6.28 | (4.55, 8.66) | 4.57 x 10^-29^ |  | 8.65 | (5.67, 13.20) | 1.52 x 10^-23^ |
| **Lateral femur** |  |  |  |  |  |  |  |  |  |  |  |
| grade 1 | 3.09 | (2.14, 4.45) | 1.50 x 10^-09^ |  | 5.36 | (3.50, 8.20) | 1.10 x 10^-14^ |  | 6.75 | (3.96, 11.48) | 2.03 x 10^-12^ |
| grade 2 | 3.51 | (2.21, 5.56) | 9.48 x 10^-08^ |  | 4.12 | (2.34, 7.27) | 9.85 x 10^-07^ |  | 5.21 | (2.44, 11.10) | 1.92 x 10^-05^ |
| grade 3 | 3.73 | (2.45, 5.66) | 7.39 x 10^-10^ |  | 9.69 | (6.27, 14.98) | 1.78 x 10^-24^ |  | 12.25 | (7.49, 20.02) | 1.66 x 10^-23^ |
| **Medial tibia** |  |  |  |  |  |  |  |  |  |  |  |
| grade 1 | 4.15 | (3.53, 4.87) | <1.0 x 10^-40^ |  | 4.54 | (3.66, 5.64) | <1.0 x 10^-40^ |  | 6.54 | (4.75, 9.00) | 1.04 x 10^-30^ |
| grade 2 | 3.82 | (2.84, 5.14) | 6.54 x 10^-19^ |  | 7.33 | (5.26, 10.22) | 6.15 x 10^-32^ |  | 11.02 | (7.29, 16.66) | 5.08 x 10^-30^ |
| grade 3 | 4.94 | (3.37, 7.24) | 2.41 x 10^-16^ |  | 7.82 | (5.16, 11.86) | 3.79 x 10^-22^ |  | 11.64 | (6.99, 19.37) | 3.70 x 10^-21^ |
| **Lateral tibia** |  |  |  |  |  |  |  |  |  |  |  |
| grade 1 | 2.69 | (2.30, 3.15) | 6.15 x 10^-35^ |  | 2.94 | (2.34, 3.68) | 1.37 x 10^-20^ |  | 3.52 | (2.46, 5.03) | 5.00 x 10^-12^ |
| grade 2 | 3.08 | (2.35, 4.05) | 7.49 x 10^-16^ |  | 5.14 | (3.70, 7.14) | 1.52 x 10^-22^ |  | 7.14 | (4.63, 11.01) | 5.76 x 10^-19^ |
| grade 3 | 3.61 | (2.69, 4.86) | 1.53 x 10^-17^ |  | 4.92 | (3.44, 7.03) | 2.19 x 10^-18^ |  | 7.26 | (4.60, 11.46) | 1.70 x 10^-17^ |
| **JSN** |  |  |  |  |  |  |  |  |  |  |  |
| Grade 1 | 1.21 | (1.05, 1.41) | 0.010* |  | 1.69 | (1.35, 2.12) | 3.68 x 10^-06^* |  | 2.23 | (1.57, 3.16) | 6.64 x 10^-06^* |
| Grade 2 | 2.03 | (1.57, 2.64) | 9.17 x 10^-08^* |  | 3.37 | (2.40, 4.73) | 2.03 x 10^-12^* |  | 4.98 | (3.17, 7.84) | 3.83 x 10^-12^* |
| Grade 3 | 4.39 | (2.82, 6.83) | 5.09 x 10^-11^ |  | 7.37 | (4.53, 12.00) | 9.30 x 10^-16^* |  | 11.10 | (6.51, 18.92) | 9.59 x 10^-19^* |

Supplementary Table S10: Adjusted associations of OP grades and JSN grades with knee OA outcomes in males and females combined.

Abbreviations: CI, Confidence Interval; HES-KOA, Knee osteoarthritis based on Hospital Episodes Statistics; HR, Hazard Ratio; JSN, Joint Space Narrowing; OP, Osteophyte; OR Odds Ratio; TKR, Total Knee Replacement. *Denotes a sex-interaction term with p<0.05. Models were adjusted for age, sex, height, weight and ethnic group. n=19,595.

|  | **Pain** | | |  | **HES KOA** | | |  | **TKR** | | |
| --- | --- | --- | --- | --- | --- | --- | --- | --- | --- | --- | --- |
|  | OR | 95% CI | *p* |  | OR | 95% CI | *p* |  | HR | 95% CI | *p* |
| **Medial femur** |  |  |  |  |  |  |  |  |  |  |  |
| grade 1 | 3.63 | (2.70, 4.89) | 1.79 x 10 ^-17^ |  | 3.30 | (2.17, 5.01) | 2.11 x 10 ^-08^ |  | 5.84 | (3.12, 10.91) | 3.16 x 10 ^-08^ |
| grade 2 | 4.30 | (3.05, 6.06) | 9.05 x 10 ^-17^ |  | 5.67 | (3.74, 8.59) | 2.75 x 10 ^-16^ |  | 11.00 | (6.27, 19.31) | 6.54 x 10 ^-17^ |
| grade 3 | 6.88 | (4.54, 10.43) | 9.24 x 10 ^-20^ |  | 6.24 | (3.79, 10.26) | 5.55 x 10 ^-13^ |  | 11.38 | (5.93, 21.85) | 2.58 x 10 ^-13^ |
| **Lateral femur** |  |  |  |  |  |  |  |  |  |  |  |
| grade 1 | 4.58 | (2.22, 9.45) | 3.87 x 10 ^-05^ |  | 7.02 | (3.11, 15.85) | 2.75 x 10 ^-06^ |  | 18.38 | (8.08, 41.79) | 3.76 x 10 ^-12^ |
| grade 2 | 4.86 | (2.33, 10.13) | 2.41 x 10 ^-05^ |  | 5.04 | (2.04, 12.43) | 4.52 x 10^-04^ |  | 6.26 | (1.55, 25.33) | 0.010 |
| grade 3 | 5.10 | (2.92, 8.92) | 1.13 x 10 ^-08^ |  | 10.86 | (6.05, 19.49) | 1.35 x 10 ^-15^ |  | 15.01 | (7.33, 30.76) | 1.36 x 10 ^-13^ |
| **Medial tibia** |  |  |  |  |  |  |  |  |  |  |  |
| grade 1 | 4.71 | (3.62, 6.12) | 5.32 x 10 ^-31^ |  | 5.08 | (3.63, 7.12) | 2.71 x 10 ^-21^ |  | 10.80 | (6.75, 17.28) | 3.14 x 10 ^-23^ |
| grade 2 | 3.82 | (2.56, 5.70) | 5.51 x 10 ^-11^ |  | 9.07 | (5.90, 13.97) | 1.18 x 10 ^-23^ |  | 17.38 | (10.03, 30.12) | 2.46 x 10 ^-24^ |
| grade 3 | 7.90 | (4.73, 13.18) | 2.63 x 10 ^-15^ |  | 7.88 | (4.41, 14.08) | 3.32 x 10 ^-12^ |  | 19.39 | (9.74, 38.62) | 3.32 x 10 ^-17^ |
| **Lateral tibia** |  |  |  |  |  |  |  |  |  |  |  |
| grade 1 | 2.72 | (2.11, 3.49) | 7.07 x 10 ^-15^ |  | 2.94 | (2.07, 4.19) | 2.22 x 10 ^-09^ |  | 4.39 | (2.50, 7.69) | 2.41 x 10 ^-07^ |
| grade 2 | 3.48 | (2.28, 5.31) | 7.33 x 10 ^-09^ |  | 5.83 | (3.56, 9.56) | 2.57 x 10 ^-12^ |  | 10.53 | (5.49, 20.20) | 1.44 x 10 ^-12^ |
| grade 3 | 5.10 | (3.37, 7.73) | 1.52 x 10 ^-14^ |  | 6.95 | (4.29, 11.25) | 3.32 x 10 ^-15^ |  | 11.40 | (6.11, 21.29) | 2.16 x 10 ^-14^ |
| **JSN** |  |  |  |  |  |  |  |  |  |  |  |
| Grade 1 | 2.28 | (1.76, 2.93) | 2.35 x 10 ^-10^ |  | 2.81 | (1.98, 3.99) | 6.22 x 10 ^-09^ |  | 4.82 | (2.92, 7.96) | 7.72 x 10 ^-10^ |
| Grade 2 | 3.44 | (2.08, 5.69) | 1.46 x 10 ^-06^ |  | 9.22 | (5.45, 15.59) | 1.15 x 10 ^-16^ |  | 11.14 | (5.42, 22.87) | 5.28 x 10 ^-11^ |
| Grade 3 | 5.48 | (2.79, 10.78) | 8.13 x 10 ^-07^ |  | 4.33 | (1.78, 10.51) | 1.21 x 10 ^-03^ |  | 5.00 | (1.23, 20.25) | 0.024 |

Supplementary Table S11: Unadjusted associations of OP grades and JSN grades with knee OA outcomes in males.

Abbreviations: CI, Confidence Interval; HES-KOA, Knee osteoarthritis based on Hospital Episodes Statistics; HR, Hazard Ratio; JSN, Joint Space Narrowing; OP, Osteophyte; OR Odds Ratio; TKR, Total Knee Replacement. n=9,449.

|  | **Pain** | | |  | **HES KOA** | | |  | **TKR** | | |
| --- | --- | --- | --- | --- | --- | --- | --- | --- | --- | --- | --- |
|  | OR | 95% CI | *p* |  | OR | 95% CI | *p* |  | HR | 95% CI | *p* |
| *Categorical* |  |  |  |  |  |  |  |  |  |  |  |
| **Medial femur** |  |  |  |  |  |  |  |  |  |  |  |
| grade 1 | 3.35 | (2.48, 4.52) | 2.97 x 10^-15^ |  | 2.86 | (1.87, 4.36) | 1.08 x 10^-06^ |  | 4.83 | (2.58, 9.06) | 8.97 x 10^-07^ |
| grade 2 | 4.00 | (2.82, 5.67) | 8.06 x 10^-15^ |  | 4.80 | (3.14, 7.32) | 3.59 x 10^-13^ |  | 8.54 | (4.84, 15.08) | 1.44 x 10^-13^ |
| grade 3 | 6.06 | (3.97, 9.23) | 5.70 x 10^-17^ |  | 4.76 | (2.86, 7.90) | 1.68 x 10^-09^ |  | 7.24 | (3.70, 14.16) | 7.14 x 10^-09^ |
| **Lateral femur** |  |  |  |  |  |  |  |  |  |  |  |
| grade 1 | 4.20 | (2.01, 8.76) | 1.29 x 10^-04^ |  | 5.63 | (2.46, 12.90) | 4.32 x 10^-05^ |  | 12.17 | (5.30, 27.90) | 3.63 x 10^-09^ |
| grade 2 | 3.66 | (1.73, 7.76) | 6.88 x 10^-04^ |  | 3.34 | (1.33, 8.39) | 1.03 x 10^-02^ |  | 3.59 | (0.88, 14.71) | 0.076 |
| grade 3 | 4.61 | (2.62, 8.13) | 1.21 x 10^-07^ |  | 9.37 | (5.17, 16.99) | 1.70 x 10^-13^ |  | 11.39 | (5.53, 23.48) | 4.30 x 10^-11^ |
| **Medial tibia** |  |  |  |  |  |  |  |  |  |  |  |
| grade 1 | 4.35 | (3.33, 5.68) | 2.29 x 10^-27^ |  | 4.37 | (3.10, 6.14) | 2.60 x 10^-17^ |  | 8.72 | (5.42, 14.03) | 4.62 x 10^-19^ |
| grade 2 | 3.42 | (2.27, 5.14) | 3.35 x 10^-09^ |  | 7.49 | (4.82, 11.62) | 2.78 x 10^-19^ |  | 13.09 | (7.48, 22.91) | 2.21 x 10^-19^ |
| grade 3 | 6.61 | (3.92, 11.14) | 1.34 x 10^-12^ |  | 5.90 | (3.25, 10.69) | 5.00 x 10^-09^ |  | 13.19 | (6.41, 27.15) | 2.48 x 10^-12^ |
| **Lateral tibia** |  |  |  |  |  |  |  |  |  |  |  |
| grade 1 | 2.54 | (1.97, 3.28) | 6.42 x 10^-13^ |  | 2.54 | (1.78, 3.64) | 3.10 x 10^-07^ |  | 3.53 | (2.01, 6.21) | 1.18 x 10^-05^ |
| grade 2 | 2.98 | (1.94, 4.58) | 6.16 x 10^-07^ |  | 4.74 | (2.87, 7.84) | 1.29 x 10^-09^ |  | 7.73 | (3.98, 15.00) | 1.50 x 10^-09^ |
| grade 3 | 4.35 | (2.85, 6.65) | 1.09 x 10^-11^ |  | 5.43 | (3.32, 8.89) | 1.72 x 10^-11^ |  | 8.02 | (4.21, 15.28) | 2.54 x 10^-10^ |
| **JSN** |  |  |  |  |  |  |  |  |  |  |  |
| Grade 1 | 2.17 | (1.68, 2.81) | 4.34 x 10^-09^ |  | 2.46 | (1.72, 3.50) | 6.91 x 10^-07^ |  | 3.78 | (2.28, 6.28) | 2.80 x 10^-07^ |
| Grade 2 | 3.10 | (1.86, 5.19) | 1.59 x 10^-05^ |  | 7.76 | (4.52, 13.31) | 9.89 x 10^-14^ |  | 8.39 | (4.05, 17.39) | 1.37 x 10^-08^ |
| Grade 3 | 5.20 | (2.62, 10.32) | 2.48 x 10^-06^ |  | 3.68 | (1.50, 9.02) | 0.004 |  | 3.67 | (0.90, 14.92) | 0.071 |

Supplementary Table S12: Adjusted associations of OP grades and JSN grades with knee OA outcomes in males.

Abbreviations: CI, Confidence Interval; HES-KOA, Knee osteoarthritis based on Hospital Episodes Statistics; HR, Hazard Ratio; JSN, Joint Space Narrowing; OP, Osteophyte; OR Odds Ratio; TKR, Total Knee Replacement. Models were adjusted for age, sex, height, weight and ethnic group. n=9,449.

|  | **Pain** | | |  | **HES KOA** | | |  | **TKR** | | |
| --- | --- | --- | --- | --- | --- | --- | --- | --- | --- | --- | --- |
|  | OR | 95% CI | *p* |  | OR | 95% CI | *p* |  | HR | 95% CI | *p* |
| **Medial femur** |  |  |  |  |  |  |  |  |  |  |  |
| grade 1 | 2.74 | (2.25, 3.34) | 1.26 x 10^-23^ |  | 3.04 | (2.22, 4.17) | 5.25 x 10^-12^ |  | 3.34 | (1.96, 5.68) | 9.18 x 10^-06^ |
| grade 2 | 5.67 | (4.35, 7.38) | 8.54 x 10^-38^ |  | 6.98 | (4.93, 9.88) | 6.54 x 10^-28^ |  | 9.10 | (5.55, 14.93) | 2.36 x 10^-18^ |
| grade 3 | 6.15 | (4.34, 8.71) | 1.65 x 10^-24^ |  | 10.99 | (7.34, 16.45) | 2.92 x 10^-31^ |  | 15.04 | (8.83, 25.63) | 2.01E x 10^23^ |
| **Lateral femur** |  |  |  |  |  |  |  |  |  |  |  |
| grade 1 | 3.36 | (2.23, 5.08) | 8.04 x 10^-09^ |  | 7.18 | (4.43, 11.66) | 1.45 x 10^-15^ |  | 8.27 | (4.20, 16.30) | 1.02 x 10^-09^ |
| grade 2 | 4.12 | (2.33, 7.31) | 1.23 x 10^-06^ |  | 6.53 | (3.24, 13.18) | 1.64 x 10^-07^ |  | 9.43 | (3.85, 23.07) | 9.02 x 10^-07^ |
| grade 3 | 3.99 | (2.19, 7.26) | 6.06 x 10^-06^ |  | 15.46 | (8.39, 28.50) | 1.65 x 10^-18^ |  | 23.96 | (12.55, 45.76) | 6.44 x 10^-22^ |
| **Medial tibia** |  |  |  |  |  |  |  |  |  |  |  |
| grade 1 | 4.62 | (3.79, 5.62) | <1.0 x 10^-40^ |  | 5.95 | (4.53, 7.82) | 1.11 x 10^-37^ |  | 7.30 | (4.81, 11.10) | 1.19 x 10^-20^ |
| grade 2 | 5.07 | (3.33, 7.72) | 4.24 x 10^-14^ |  | 9.71 | (5.92, 15.91) | 1.90 x 10^-19^ |  | 14.97 | (8.20, 27.34) | 1.24 x 10^-18^ |
| grade 3 | 5.08 | (2.94, 8.78) | 5.52 x 10^-09^ |  | 18.19 | (10.32, 32.06) | 1.14 x 10^-23^ |  | 23.35 | (11.75, 46.42) | 2.56 x 10^-19^ |
| **Lateral tibia** |  |  |  |  |  |  |  |  |  |  |  |
| grade 1 | 3.18 | (2.61, 3.86) | 5.77 x 10^-31^ |  | 4.08 | (3.05, 5.44) | 1.43 x 10^-21^ |  | 4.73 | (3.00, 7.47) | 2.52 x 10^-11^ |
| grade 2 | 3.83 | (2.70, 5.41) | 3.63 x 10^-14^ |  | 7.49 | (4.90, 11.44) | 1.19 x 10^-20^ |  | 10.49 | (5.98, 18.39) | 2.45 x 10^-16^ |
| grade 3 | 3.87 | (2.59, 5.77) | 3.47 x 10^-11^ |  | 6.81 | (4.13, 11.22) | 5.50 x 10^-14^ |  | 12.12 | (6.49, 22.66) | 5.30 x 10^-15^ |
| **JSN** |  |  |  |  |  |  |  |  |  |  |  |
| Grade 1 | 1.00 | (0.84, 1.20) | 0.960 |  | 1.49 | (1.12, 1.97) | 0.005 |  | 1.84 | (1.15, 2.92) | 0.011 |
| Grade 2 | 1.90 | (1.41, 2.56) | 2.24 x 10^-05^ |  | 2.67 | (1.71, 4.15) | 1.46 x 10^-05^ |  | 5.38 | (3.06, 9.43) | 4.57 x 10^-09^ |
| Grade 3 | 5.07 | (2.90, 8.87) | 1.28 x 10^-08^ |  | 15.98 | (8.93, 28.60) | 9.93 x 10^-21^ |  | 28.97 | (16.21, 51.80) | 6.81 x 10^-30^ |

Supplementary Table S13: Unadjusted associations of OP grades and JSN grades with knee OA outcomes in females.

Abbreviations: CI, Confidence Interval; HES-KOA, Knee osteoarthritis based on Hospital Episodes Statistics; HR, Hazard Ratio; JSN, Joint Space Narrowing; OP, Osteophyte; OR Odds Ratio; TKR, Total Knee Replacement. n=10,146.

|  | **Pain** | | |  | **HES KOA** | | |  | **TKR** | | |
| --- | --- | --- | --- | --- | --- | --- | --- | --- | --- | --- | --- |
|  | OR | 95% CI | *p* |  | OR | 95% CI | *p* |  | HR | 95% CI | *p* |
| **Medial femur** |  |  |  |  |  |  |  |  |  |  |  |
| grade 1 | 2.40 | (1.96, 2.93) | 2.51 x 10^-17^ |  | 2.40 | (1.74, 1.74) | 1.03 x 10^-07^ |  | 2.38 | (1.39, 4.08) | 0.002 |
| grade 2 | 4.74 | (3.61, 6.21) | 3.32 x 10^-29^ |  | 5.08 | (3.55, 3.55) | 6.10 x 10^-19^ |  | 5.75 | (3.46, 9.55) | 1.46 x 10^-11^ |
| grade 3 | 4.77 | (3.33, 6.83) | 1.78 x 10^-17^ |  | 7.60 | (5.00, 5.00) | 2.84 x 10^-21^ |  | 9.50 | (5.48, 16.46) | 1.03 x 10^-15^ |
| **Lateral femur** |  |  |  |  |  |  |  |  |  |  |  |
| grade 1 | 2.74 | (1.79, 4.19) | 3.13 x 10^-06^ |  | 5.15 | (3.12, 3.12) | 1.30 x 10^-10^ |  | 5.07 | (2.55, 10.09) | 3.82 x 10^-06^ |
| grade 2 | 3.39 | (1.89, 6.09) | 4.43 x 10^-05^ |  | 4.85 | (2.37, 2.37) | 1.55 x 10^-05^ |  | 6.38 | (2.59, 15.70) | 5.45 x 10^-05^ |
| grade 3 | 2.86 | (1.53, 5.34) | 0.001 |  | 9.80 | (5.14, 5.14) | 4.07 x 10^-12^ |  | 12.29 | (6.22, 24.29) | 5.35 x 10^-13^ |
| **Medial tibia** |  |  |  |  |  |  |  |  |  |  |  |
| grade 1 | 4.01 | (3.27, 4.91) | <1.0 x 10^-40^ |  | 4.63 | (3.50, 3.50) | 9.21 x 10^-27^ |  | 5.19 | (3.38, 7.95) | 4.44 x 10^-14^ |
| grade 2 | 4.34 | (2.82, 6.68) | 2.50 x 10^-11^ |  | 7.15 | (4.31, 4.31) | 2.77 x 10^-14^ |  | 9.11 | (4.93, 16.84) | 1.75 x 10^-12^ |
| grade 3 | 3.52 | (1.99, 6.22) | 1.45 x 10^-05^ |  | 10.49 | (5.79, 5.79) | 9.08 x 10^-15^ |  | 10.61 | (5.17, 21.78) | 1.21 x 10^-10^ |
| **Lateral tibia** |  |  |  |  |  |  |  |  |  |  |  |
| grade 1 | 2.77 | (2.27, 3.39) | 3.50 x 10^-23^ |  | 3.20 | (2.38, 2.38) | 1.55 x 10^-14^ |  | 3.41 | (2.14, 5.42) | 2.21 x 10^-07^ |
| grade 2 | 3.14 | (2.20, 4.48) | 3.25 x 10^-10^ |  | 5.45 | (3.53, 3.53) | 2.21 x 10^-14^ |  | 6.76 | (3.81, 11.99) | 6.44 x 10^-11^ |
| grade 3 | 3.05 | (2.01, 4.61) | 1.38 x 10^-07^ |  | 4.41 | (2.62, 2.62) | 2.22 x 10^-08^ |  | 6.65 | (3.48, 12.71) | 9.50 x 10^-09^ |
| **JSN** |  |  |  |  |  |  |  |  |  |  |  |
| Grade 1 | 0.96 | (0.80, 1.15) | 0.665 |  | 1.37 | (1.03, 1.03) | 0.032 |  | 1.61 | (1.01, 2.57) | 0.045 |
| Grade 2 | 1.74 | (1.28, 2.36) | 3.61 x 10^-04^ |  | 2.13 | (1.35, 1.35) | 0.001 |  | 3.85 | (2.18, 6.82) | 3.65 x 10^-06^ |
| Grade 3 | 3.76 | (2.11, 6.70) | 7.28 x 10^-06^ |  | 10.20 | (5.55, 5.55) | 7.32 x 10^-14^ |  | 15.45 | (8.47, 28.16) | 4.17 x 10^-19^ |

Supplementary Table S14: Adjusted associations of OP grades and JSN grades with knee OA outcomes in females.

Abbreviations: CI, Confidence Interval; HES-KOA, Knee osteoarthritis based on Hospital Episodes Statistics; HR, Hazard Ratio; JSN, Joint Space Narrowing; OP, Osteophyte; OR Odds Ratio; TKR, Total Knee Replacement. Models were adjusted for age, height, weight and ethnic group. n=10,146.

|  | Unadjusted | | | | | | | | | | |
| --- | --- | --- | --- | --- | --- | --- | --- | --- | --- | --- | --- |
|  | **Pain** | | |  | **HES KOA** | | |  | **TKR** | | |
| **rKOA exposure** | OR | 95% CI | *p* |  | OR | 95% CI | *p* |  | HR | 95% CI | *p* |
|  | **All** | | | | | | | | | | |
| Grade 1 | 2.09 | (1.89, 2.31) | <1.0 x 10^-40^* |  | 2.68 | (2.27, 3.15) | 3.29 x 10^-32^ |  | 4.45 | (3.28, 6.03) | 5.37 x 10^-22^ |
| Grade 2 | 3.87 | (3.29, 4.55) | <1.0 x 10^-40^ |  | 7.75 | (6.30, 9.52) | <1.0 x 10^-40^* |  | 15.75 | (11.43, 21.71) | <1.0 x 10^-40^* |
| Grades 3-4 | 8.62 | (6.63, 11.21) | <1.0 x 10^-40^ |  | 13.40 | (9.97, 18.01) | <1.0 x 10^-40^* |  | 32.58 | (22.50, 47.17) | <1.0 x 10^-40^* |
|  | **Male** | | | | | | | | | | |
| Grade 1 | 2.73 | (2.31, 3.21) | 4.10 x 10^-33^ |  | 3.44 | (2.71, 4.37) | 3.91 x 10 ^-24^ |  | 5.90 | (3.81, 9.13) | 1.83 x 10^-15^ |
| Grade 2 | 4.53 | (3.43, 6.00) | 2.67 x 10^-26^ |  | 11.51 | (8.45, 15.68) | <1.0 x 10^-40^ |  | 24.54 | (15.84, 38.02) | <1.0 x 10^-40^ |
| Grades 3-4 | 8.89 | (6.02, 13.13) | 5.21 x 10^-28^ |  | 7.37 | (4.58, 11.87) | 2.07 x 10-^16^ |  | 18.44 | (9.86, 34.47) | 6.79 x 10^-20^ |
|  | **Female** | | | | | | | | | | |
| Grade 1 | 1.87 | (1.64, 2.13) | 5.75 x 10^-21^ |  | 2.70 | (2.13, 3.40) | 7.80 x 10^-17^ |  | 4.04 | (2.63, 6.21) | 2.10 x 10^-10^ |
| Grade 2 | 3.66 | (2.99, 4.47) | 1.59 x 10^-36^ |  | 7.09 | (5.31, 9.46) | <1.0 x 10^-40^ |  | 12.19 | (7.59, 19.58) | 4.17 x 10^-25^ |
| Grades 3-4 | 8.48 | (5.94, 12.09) | 4.64 x 10^-32^ |  | 23.09 | (15.62, 34.14) | <1.0 x 10^-40^ |  | 49.16 | (30.31, 79.71) | <1.0 x 10^-40^ |
|  | Adjusted | | | | | | | | | | |
|  | **All** | | | | | | | | | | |
| Grade 1 | 2.04 | (1.84, 2.26) | <1.0 x 10^-40^* |  | 2.67 | (2.25, 3.16) | 1.47 x 10^-29^ |  | 3.97 | (2.90, 5.42) | 5.64 x 10^-18^ |
| Grade 2 | 3.65 | (3.08, 4.31) | <1.0 x 10^-40^ |  | 7.26 | (5.85, 9.00) | <1.0 x 10^-40^* |  | 12.87 | (9.22, 17.96) | <1.0 x 10^-40^* |
| Grades 3-4 | 7.08 | (5.41, 9.27) | <1.0 x 10^-40^ |  | 10.24 | (7.53, 13.93) | <1.0 x 10^-40^* |  | 21.11 | (14.28, 31.19) | <1.0 x 10^-40^* |
|  | **Male** | | | | | | | | | | |
| Grade 1 | 2.62 | (2.22, 3.10) | 1.20 x 10^-29^ |  | 3.07 | (2.41, 3.91) | 1.46 x 10^-19^ |  | 4.90 | (3.14, 7.63) | 2.33 x 10^-12^ |
| Grade 2 | 4.34 | (3.27, 5.76) | 3.65 x 10^-24^ |  | 10.06 | (7.35, 13.78) | <1.0 x 10^-40^ |  | 19.61 | (12.55, 30.65) | <1.0 x 10^-40^ |
| Grades 3-4 | 7.59 | (5.10, 11.29) | 1.65 x 10^-23^ |  | 5.71 | (3.50, 9.30) | 2.65 x 10^-12^ |  | 12.62 | (6.59, 24.17) | 2.13E^-14^ |
|  | **Female** | | | | | | | | | | |
| Grade 1 | 1.74 | (1.52, 1.99) | 3.61 x 10^-16^ |  | 2.34 | (1.84, 2.96) | 2.51 x 10^-12^ |  | 3.28 | (2.12, 5.07) | 9.14 x 10^-08^ |
| Grade 2 | 3.22 | (2.61, 3.97) | 3.90 x 10^-28^ |  | 5.58 | (4.14, 7.53) | 1.74 x 10^-29^ |  | 8.75 | (5.39, 14.22) | 1.96 x 10^-18^ |
| Grades 3-4 | 6.53 | (4.52, 9.43) | 1.66 x 10^-23^ |  | 15.52 | (10.31, 23.35) | <1.0 x 10^-40^ |  | 28.63 | (17.09, 47.96) | 3.33 x 10^-37^ |

Supplementary Table S15: Association of radiographic knee osteoarthritis (rKOA) grades with clinical outcomes, overall and stratified by sex.

Abbreviations: CI, Confidence Interval; HES-KOA, Knee Osteoarthritis Based on Hospital Episodes Statistics; HR, Hazard Ratio; JSN, Joint Space Narrowing; OP, Osteophyte; OR Odds Ratio; TKR, Total Knee Replacement. *Denotes a sex-interaction term with p<0.05. Models were adjusted for age, sex, height, weight and ethnic group. Overall, n=19,595; males, n=9449; females n=10,146

|  | **Total** | **Male** | **Female** |
| --- | --- | --- | --- |
|  | N=19595 | N=9449 | N=10146 |
| **rKOA grade** |  |  |  |
| 0 | 14895 (76.01%) | 8366 (88.54%) | 6529 (64.35%) |
| 1 | 3312 (16.90%) | 787 (8.33%) | 2525 (24.89%) |
| 2 | 1128 (5.76%) | 198 (2.10%) | 930 (9.17%) |
| 3 | 182 (0.93%) | 69 (0.73%) | 113 (1.11%) |
| 4 | 78 (0.40%) | 29 (0.31%) | 49 (0.48%) |

Supplementary Table S16: Prevalence of height-adjusted radiographic knee osteoarthritis (rKOA)

Radiographic knee osteoarthritis (rKOA) grades were generated based on a combination of osteophyte grades and joint space narrowing (JSN) grades. In the sensitivity analysis, the minimum joint space width (mJSW) of the medial compartment, which was used to define JSN grade, was normalised by the mean height of the population to account for height-related variations in mJSW.

|  | **Pain** | | | **HES-KOA** | | | **TKR** | | |
| --- | --- | --- | --- | --- | --- | --- | --- | --- | --- |
|  | OR | lower CI | *p* | OR | lower CI | *p* | HR | lower CI | *p* |
| **All** | | | | | | | | | |
| Grade 1 | 1.93 | (1.73, 2.14) | 2.05 x 10^-34^* | 2.69 | (2.26, 3.20) | 3.18 x 10^-29^* | 3.47 | (2.51, 4.80) | 4.61 x 10^-14^* |
| Grade 2 | 2.61 | (2.24, 3.03) | 4.09 x 10^-35^* | 5.08 | (4.09, 6.32) | <1.0 x 10^-40^* | 9.19 | (6.51, 12.98) | 2.04 x 10^-36^* |
| Grades 3-4 | 7.40 | (5.72, 9.58) | <1.0 x 10^-40^ | 10.67 | (7.90, 14.39) | <1.0 x 10^-40^* | 19.72 | (13.40, 29.02) | <1.0 x 10^-40^ |
| Grade >1 | 2.28 | (2.08, 2.50) | <1.0 x 10^-40^* | 3.62 | (3.12, 4.21) | <1.0 x 10^-40^* | 5.86 | (4.47, 7.69) | 1.76 x 10^-37^* |
| **Males** | | | | | | | | | |
| Grade 1 | 2.81 | (2.37, 3.33) | 1.95 x 10^-32^ | 3.91 | (3.09, 4.95) | 6.95 x 10^-30^ | 5.78 | (3.76, 8.88) | 1.34 x 10^-15^ |
| Grade 2 | 4.40 | (3.27, 5.92) | 1.36 x 10^-22^ | 9.18 | (6.57, 12.85) | 2.22 x 10^-38^ | 18.63 | (11.74, 29.56) | 2.33 x 10^-35^ |
| Grades 3-4 | 7.47 | (4.95, 11.26) | 8.72 x 10^-22^ | 5.26 | (3.14, 8.81) | 3.05 x 10^-10^ | 12.06 | (6.16, 23.61) | 3.74 x 10^-13^ |
| Grade >1 | 3.37 | (2.91, 3.90) | <1.0 x 10^-40^ | 4.88 | (3.99, 5.96) | <1.0 x 10^-40^ | 8.67 | (6.06, 12.41) | 3.10 x 10^-32^ |
| **Females** | | | | | | | | | |
| Grade 1 | 1.56 | (1.36, 1.78) | 5.54 x 10^-11^ | 1.85 | (1.45, 2.37) | 7.97 x 10^-07^ | 2.02 | (1.27, 3.22) | 0.003 |
| Grade 2 | 2.11 | (1.76, 2.53) | 5.96 x 10^-16^ | 3.40 | (2.55, 4.53) | 9.29 x 10^-17^ | 5.14 | (3.19, 8.29) | 1.94 x 10^-11^ |
| Grades 3-4 | 6.90 | (4.95, 9.63) | 5.65 x 10^-30^ | 13.93 | (9.49, 20.45) | <1.0 x 10^-40^ | 21.96 | (13.15, 36.66) | 3.36 x 10^-32^ |
| Grade >1 | 1.83 | (1.62, 2.05) | 9.71 x 10^-24^ | 2.63 | (2.13, 3.25) | 2.11 x 10^-19^ | 3.66 | (2.50, 5.37) | 2.85 x 10^-11^ |

Supplementary Table S17: Results of the sensitivity analysis looking at the association of height-adjusted rKOA grades with clinical outcomes.

This table presents the regression results for the association of height-adjusted radiographic knee osteoarthritis (rKOA) grades with knee osteoarthritis outcomes. The outcomes evaluated include prolonged knee pain, hospital-diagnosed knee OA (HES-KOA), and subsequent total knee replacement (TKR). Analyses were adjusted for age, sex (in combined analysis), height, weight and ethnic category. *Denotes a sex-interaction term with p<0.05.

# Supplementary Data S1. Supplementary Methods

## Assessment of covariates

The selection of covariates was predetermined based on existing literature associating these variables with knee osteoarthritis risk. Height and weight measurements were taken at the time of the DXA scan, following standardized procedures, while age and sex were self-reported during recruitment. Participants self-reported their ethnicity, which was then categorized into groups including White, Black, Asian, Chinese, Mixed-Heritage and Other.

## Generation of a rKOA osteophyte grades

In this study, osteophyte area cut-offs were generated using a combination of manually graded osteophyte grades and shading of osteophyte area. Binary variables were first created for Grade 2 and Grade 3 osteophytes in both the medial and lateral femur regions. These variables were assigned a value of 0 if the manually graded osteophyte was below the specified grade and a value of 1 if the manually graded osteophyte matched the specified grade. Optimal cut-points for osteophyte area were then estimated using the Youden Index method, which determines the optimal threshold for classification based on a continuous predictor variable, by maximizing the sum of sensitivity and specificity. The reference variable was the binary outcome variable, while the classification variable was the continuous osteophyte area measurement. The empirical optimal cut-points for Grade 2 and Grade 3 osteophytes were as follows: medial femur (grade 2: 14.72 mm^2^, grade 3: 25.78 mm), lateral femur (14.03 mm^2^, 24.62 mm^2^), medial tibia (12.20 mm^2^, 20.28 mm^2^), and lateral tibia (10.53 mm^2^, 18.65 mm^2^). Based on these cut-offs, osteophyte grades were determined accordingly:

|  | **Medial Femur** | | **Lateral Femur** | | **Medial Tibia** | | **Lateral Tibia** | |
| --- | --- | --- | --- | --- | --- | --- | --- | --- |
| Grade | lower | upper | lower | upper | lower | upper | lower | upper |
| 1 | >0 mm^2^ | ≤15 mm^2^ | >0 mm^2^ | ≤14 mm^2^ | >0 mm^2^ | ≤12 mm^2^ | >0 mm^2^ | ≤11 mm^2^ |
| 2 | ≥15 mm^2^ | <26 mm^2^ | ≥14 mm^2^ | <25 mm^2^ | ≥12 mm^2^ | ≤20 mm^2^ | ≥11 mm^2^ | ≤19 mm^2^ |
| 3 | ≥26 mm^2^ |  | ≥25 mm^2^ |  | ≥20 mm^2^ |  | ≥19 mm^2^ |  |

These grades were then multiplied by 0.5, summed (with a maximum value of 6), and added to the JSN grade (which has a maximum value of 3) to derive a combined score, with a maximum value of 9. Subsequently, the overall rKOA grade was determined based on this combined score, as detailed in the main text.

## Sensitivity analysis

Height normalisation of medial joint space width (mJSW) was performed to mitigate potential confounding effects of participant height on mJSW measurements. Initially, a linear regression analysis was conducted using height as the independent variable and mJSW as the dependent variable. This regression provided a beta coefficient representing the change in mJSW per unit change in height. Subsequently, for each participant, the difference between their height and the mean height of the study population was calculated. This difference was multiplied by the beta coefficient to derive an inflation factor, which served to adjust each individual's mJSW measurement based on their height deviation from the mean. Adjustments were made by adding the inflation factor for individuals taller than the mean height and subtracting it for those shorter. These normalised mJSW values were then used to redefine Joint Space Narrowing (JSN) grades as before (JSN grade 0 for mJSW >3mm, grade 1 for mJSW >2.5mm and <3mm, grade 2 for mJSW >2mm and <2.5mm, and grade 3 for mJSW <2mm). The revised JSN grades were integrated with osteophyte grades to compute overall radiographic knee osteoarthritis (rKOA) grade, consistent with the methodology detailed in the main text. This approach ensured that mJSW measurements accurately reflected joint space narrowing independent of height variations.
